# Polyunsaturated Fatty Acids and Human Health: A Key to Modern Nutritional Balance in Association with Polyphenolic Compounds from Food Sources

**DOI:** 10.3390/foods14010046

**Published:** 2024-12-27

**Authors:** Magdalena Mititelu, Dumitru Lupuliasa, Sorinel Marius Neacșu, Gabriel Olteanu, Ștefan Sebastian Busnatu, Andreea Mihai, Violeta Popovici, Nicoleta Măru, Steluța Constanța Boroghină, Sebastian Mihai, Corina-Bianca Ioniță-Mîndrican, Alexandru Scafa-Udriște

**Affiliations:** 1 Department of Clinical Laboratory and Food Safety, Faculty of Pharmacy, “Carol Davila” University of Medicine and Pharmacy, 020956 Bucharest, Romania; magdalena.mititelu@umfcd.ro; 2 Department of Pharmaceutical Technology and Bio-Pharmacy, Faculty of Pharmacy, Carol Davila University of Medicine and Pharmacy, 020945 Bucharest, Romania; dumitru.lupuliasa@umfcd.ro; 3 Department of Cardio-Thoracic Pathology, Faculty of Medicine, “Carol Davila” University of Medicine and Pharmacy, 050474 Bucharest, Romania; stefan.busnatu@umfcd.ro (Ș.S.B.); alexandru.scafa@umfcd.ro (A.S.-U.); 4 Department of Pulmonology, University of Medicine and Pharmacy of Craiova, 200349 Craiova, Romania; andreea.mihai@umfcv.ro; 5 “Costin C. Kiriţescu” National Institute of Economic Research—Center for Mountain Economics (INCE-CEMONT) of Romanian Academy, 725700 Vatra-Dornei, Romania; violeta.popovici@ce-mont.ro; 6 Department of Anatomy, Faculty of Dental Medicine, “Carol Davila” University of Medicine and Pharmacy, 020945 Bucharest, Romania; nicoleta.maru@umfcd.ro; 7 Department of Complementary Sciences, History of Medicine and Medical Culture, Faculty of Medicine, “Carol Davila” University of Medicine and Pharmacy, 050474 Bucharest, Romania; steluta.boroghina@umfcd.ro; 8 Department of Therapeutic Chemistry, Faculty of Pharmacy, “Ovidius“ University of Constanta, 6 Căpitan Aviator Al Șerbănescu Street, 900470 Constanta, Romania; sebastian.mihai@univ-ovidius.ro; 9 Department of Toxicology, Faculty of Pharmacy, “Carol Davila” University of Medicine and Pharmacy, 020945 Bucharest, Romania; corina-bianca.ionita-mindrican@drd.umfcd.ro

**Keywords:** monounsaturated fatty acids, polyunsaturated fatty acids, saturated fats, metabolic syndrome, essential fatty acids, marine lipids, polyphenols

## Abstract

Polyunsaturated fatty acids (PUFAs) are vital dietary elements that play a significant role in human nutrition. They are highly regarded for their positive contributions to overall health and well-being. Beyond the fact that they provide a substantial supply of energy to the body (a role that saturated fats can also perform), these unsaturated fatty acids and, especially, the essential ones are involved in cell membrane structure, blood pressure regulation, and coagulation; participate in the proper functioning of the immune system and assimilation of fat-soluble vitamins; influence the synthesis of pro- and anti-inflammatory substances; and protect the cardiovascular system. Modern diets like the Western diet and the American diet are rich in saturated fats found especially in fast food products, sweets, and processed foods, a fact that has led to an increase in the prevalence of metabolic diseases worldwide (obesity, type II diabetes, gout, cardiovascular disease). Nutritionists have drawn attention to the moderate consumption of saturated fats and the need to increase the intake of unsaturated fats to the detriment of saturated ones. This paper examines the biochemical roles of polyunsaturated fats, particularly essential fatty acids, and contrasts their benefits with the detrimental effects of saturated fat overconsumption. Furthermore, it highlights the necessity for dietary shifts towards increased PUFA intake to mitigate the global burden of diet-related health issues. The co-occurrence of PUFAs and polyphenols in plant-based foods highlights the sophistication of nature’s design. These bioactive compounds are not randomly distributed but are present in foods humans have consumed together historically. From traditional diets like the Mediterranean, which pairs olive oil (PUFAs and polyphenols) with vegetables and legumes, to Asian cuisines combining sesame seeds with turmeric, cultural practices have long harnessed this natural synergy.

## 1. Introduction

Lipids are basic structures for cells, tissues, and organs, with major importance for synthesizing some active compounds [1]. The fatty acids that participate in the formation of lipids are divided into two main categories: saturated fatty acids that do not present double carbon–carbon bonds in the structure of the hydrocarbon chain and unsaturated fatty acids, those that present one or more double bonds [2].

**Figure 1:**
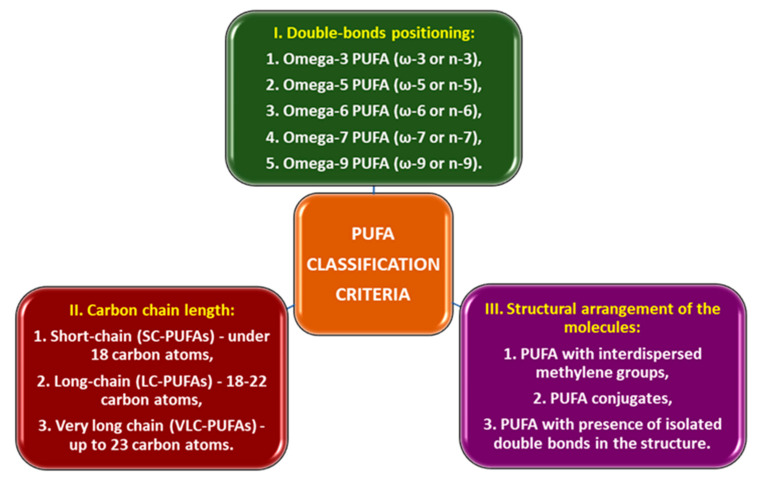
PUFA classification based on several criteria.

Polyunsaturated fatty acids (PUFAs) contain two or more double bonds with cis configurations in their hydrocarbon chain structure. PUFAs can be classified according to several structural criteria (Figure 1) [2,3,4]:

PUFAs are integral components of all body membranes. They are embedded within phospholipids and other complex lipids, where they are esterified to glycerol or other polyols. These lipids often have hydrophilic molecules attached, which contribute to their structural and functional roles in cellular membranes [4,5,6].

The polyunsaturated fatty acids α-linolenic acid (ALA, ω-3) and linoleic acid (LA, ω-6) are classified as essential fatty acids because the human body cannot synthesize them, and they must be obtained through the diet. Other polyunsaturated fatty acids, such as eicosapentaenoic acid (EPA) and docosahexaenoic acid (DHA), are not strictly essential, as they can be synthesized in limited amounts from their essential precursors [2].

To be biologically active, the double bonds of PUFAs must be in the cis configuration. These give the molecules a rounded shape. The methylene-bridged polyunsaturated structure makes rotation around adjacent C-C single bonds more favorable compared to the lack of double bonds, significantly increasing the flexibility of the molecules. The “rounded” shape and the flexibility of PUFAs lower the melting point and thus increase the fluidity of plasma membranes [4].

The PUFAs ω-3 & ω-6, particularly LC-PUFA and VLC-PUFA, the representatives with a higher degree of unsaturation and the essential members derived from plant sources, play a vital role in human health, starting from the stage of conception to the stages later in development and aging, including roles in cell membrane composition, metabolism, translation and signal amplification, and gene expression. Adequate food intake of PUFAs is vital in childhood and essential in supporting the body’s growth and development. Also, an adequate food intake of ω-3 and ω-6 PUFAs is recommended to prevent some diseases, such as cardiovascular diseases, obesity, degenerative diseases, etc. [7].

This article delves into the multifaceted role of polyunsaturated fatty acids in fostering and maintaining overall health. It investigates the positive effects and potential drawbacks of PUFA intake, emphasizing their biological significance, impact on various health outcomes, and practical guidelines for achieving balanced and health-promoting dietary consumption.

## 2. Materials and Methods

### 2.1. Literature Search

A detailed literature review examined the effects of polyunsaturated fatty acids (PUFAs), particularly Omega-3 and Omega-6, on physical health, cardiovascular diseases (CVDs), depression, and other chronic conditions. The analysis involved a systematic search of major scientific databases, including ScienceDirect, Web of Science, and PubMed, utilizing focused keywords such as PUFAs, Omega-3, Omega-6, cardiovascular diseases, depression, and chronic illnesses to ensure comprehensive coverage.

### 2.2. The Search Yield

- ScienceDirect: 791 articles, predominantly in Food Chemistry (591) and Clinical Nutrition (200).
- Web of Science: 1395 open-access articles across key disciplines, including Nutrition and Dietetics (1207), Food Science and Technology (1139), Pharmacology and Pharmacy (530), Pathology (432), Cardiovascular System/Cardiology (260), Endocrinology/Metabolism (405), Neurosciences (163), and Oncology.
- PubMed: 2953 articles relevant to the search terms.

The results were screened for relevance to inform this review’s focus on the clinical and biochemical implications of PUFA consumption in the context of the specified health conditions.

## 3. Food Sources and Dietary Health Benefits

Food plays an essential role in providing necessary healthy fats. Due to their diverse roles in health and in reducing disease risk factors, the scientific interest in ω-3 and ω-6 PUFAs has increased in the recent past (Table 1). Oils abundant in PUFAs are predominantly obtained from plant-based sources and marine origins, such as fish and algae [3].

Converging polyunsaturated fatty acids (PUFAs) and polyphenolic compounds within sustainable, plant-based dietary sources embodies a powerful health and environmental synergy narrative. These bioactive nutrients, found abundantly in nuts, seeds, fruits, and certain oils, demonstrate how natural systems intricately balance nutritional needs with ecological harmony. Their biochemical interplay offers profound health benefits—reducing inflammation, enhancing antioxidant defenses, and supporting cardiovascular, neurological, and metabolic well-being.

Beyond their physiological impact, adopting diets rich in these foods reflects a pivotal shift toward sustainability. Plant-based nutrition minimizes resource depletion, fosters biodiversity, and reduces greenhouse gas emissions, directly addressing the pressing challenges of climate change and environmental degradation and aligning individual health with global ecological goals; such dietary patterns serve as a model for future food systems that are both resilient and regenerative.

ALA and LA are vital fatty acids that the human body cannot produce on its own, which means they must be obtained through the diet to fulfill physiological needs. From a clinical and therapeutic perspective, their biochemical activity leads to the production of anti-inflammatory eicosanoids, regulation of lipid metabolism, and protection of the arterial system against the development and progression of atherosclerosis [44,45]. Balancing Omega-6 and Omega-3 fatty acids is important for minimizing inflammation. Research indicates that the disproportionately high intake of Omega-6 fatty acids typical of Western diets is linked to the development of chronic inflammation [46].

ω-3 SC-PUFAs are found predominantly in oily seeds (rape, flax, chia, hemp) and fruits (walnuts, coconuts), their oil, and in more moderate amounts in some green vegetables. ω-3 LC-PUFAs (EPA, DHA) are found only in algae and marine animals that can accumulate EPA and DHA. Fatty fish from cold water, like salmon, mackerel, herring, halibut, anchovies, and sardines, are rich in EPA and DHA [47]. These marine organisms contain much higher Omega-3 acids than Omega-6, even seven times more. The problem of marine organisms that obtain Omega-3 saturated fatty acids is the danger of contamination with various pollutants from the marine environment (microplastics, pesticides, toxic metals) [48,49]. Omega-3 supplement manufacturers remove these pollutants through various processes. Terrestrial food sources can also be contaminated and endanger the safety of the consumer [50,51,52,53]. Including nuts and seeds in diets enriches them with polyunsaturated fatty acids (PUFAs) and introduces an abundance of polyphenolic compounds when paired with fruits and vegetables. This synergy amplifies health outcomes: Omega-3 PUFAs in flaxseeds or walnuts and polyphenols in berries collectively reduce systemic inflammation, mitigating risks of chronic diseases like diabetes and cardiovascular conditions; polyphenols stabilize PUFAs, preventing lipid peroxidation and ensuring their efficacy in cellular repair and brain health. For example, a breakfast bowl of chia seeds (PUFAs) with mixed berries (polyphenols) showcases this interplay, serving as a functional meal with immediate and long-term health benefits.

Fish do not synthesize fatty acids; they ingest the microalgae and bacteria that contain them. This is why farmed fish-fed partially plant-based foods contain fewer Omega-3 acids than wild fish. Many marine invertebrate organisms, such as crustaceans, mollusks, and corals, can synthesize Omega-3 acids and provide them to other marine creatures [54].

Notably, the freezing process reduces the content of polyunsaturated fatty acids. Therefore, checking the fishing dates for fish and seafood is important. Due to degradation, over a quarter of their Omega-3 content is lost after approximately 90 days of freezing [55].

Saturated fats, characterized by the absence of double bonds and a solid state at room temperature, are primarily present in animal-based foods and specific plant oils. Excessive consumption of saturated fats is strongly associated with an increased risk of cardiovascular disease, mainly due to their role in raising low-density lipoprotein (LDL) cholesterol levels. Elevated LDL-cholesterol is a well-documented risk factor for atherosclerosis, characterized by the buildup of fatty plaques within arterial walls, leading to reduced blood flow and a greater risk of heart attacks and strokes [56]. Biochemically, incorporating saturated fats into cell membranes leads to a tightly packed and less flexible lipid bilayer due to their straight-chain structure. In contrast, polyunsaturated fatty acids (PUFAs) create a more fluid and dynamic membrane environment with their multiple double bonds and kinked structure. This difference in lipid composition directly affects membrane fluidity, which is crucial for maintaining normal cellular functions, including receptor mobility, intracellular signaling, and efficient nutrient and ion transport. A rigid membrane structure induced by saturated fat accumulation can hinder the proper functioning of insulin receptors by reducing their mobility and impairing their ability to interact with signaling proteins. This biochemical disruption undermines insulin signaling pathways, contributing to insulin resistance—a critical factor in developing type 2 diabetes [57].

Moreover, the metabolism of saturated fats can produce pro-inflammatory molecules, further exacerbating the risk of chronic diseases [58]; that is why it’s essential to include foods rich in Omega-3 PUFAs with an anti-inflammatory effect [59].

The widespread consumption of processed foods, combined with the disproportionate intake of fatty acids—characterized by an excess of Omega-6 fatty acids and a deficiency of Omega-3 fatty acids—presents significant challenges to achieving a healthy nutritional balance in today’s diets. Moreover, there is a need to shift towards diets emphasizing natural sources of healthy fats, such as fatty fish, nuts, seeds, and plant oils, while reducing excessive intake of processed and Omega-6-rich foods. This approach supports physical health and aligns with a more sustainable and holistic way of eating, fostering a deeper connection between diet and long-term health outcomes in our increasingly fast-paced world [60,61].

**Figure 2:**
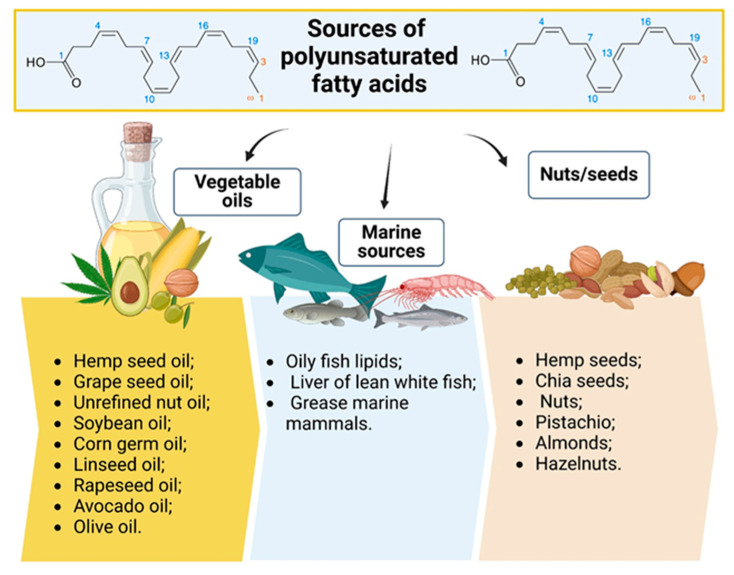
Foods rich in polyunsaturated fatty acids. Created with BioRender.com (accessed on 15 October 2024).

Increasing Omega-3 fatty acid consumption is vital to achieving a healthy balance between Omega-3 and Omega-6 fatty acids, essential for lowering the risk of chronic inflammation and associated diseases [62]. Incorporating fish with a high Omega-3 content is strongly recommended. Eating at least two servings of fish rich in Omega-3 PUFAs each week is advisable for optimal health. Plant-based foods rich in ALA can also contribute to a balanced intake [33]. These plant-based foods can be particularly valuable for individuals who follow vegetarian or vegan diets (Figure 2). In cases where dietary intake is insufficient, high-quality Omega-3 supplements, including fish oil, krill oil, or algae-derived options, may be considered to ensure adequate consumption.

Reducing Omega-6 fatty acid intake involves minimizing the consumption of processed and fried foods, which are often rich in vegetable oils such as soybean, corn, and sunflower. These oils can alter the delicate balance between Omega-3 and Omega-6 fatty acids. For a healthier alternative, oils like olive, avocado, and coconut are recommended, though they should also be consumed in moderation [63].

Prioritizing whole, unprocessed foods can also support a better balance of these fatty acids. Including diverse leafy greens, nuts, seeds, and lean proteins in the diet can contribute to maintaining this equilibrium. When selecting snacks, it is advisable to choose options like walnuts and flaxseeds, which are rich in Omega-3, while consuming Omega-6-rich nuts and seeds, such as almonds and sunflower seeds, in moderation [61].

Public health recommendations have increasingly emphasized reducing saturated fat intake in favor of polyunsaturated and monounsaturated fats to mitigate the risk of chronic diseases. Future research should continue to explore the optimal balance of fatty acid intake, considering individual variability in response to different types of dietary fats [64,65].

To include products with healthy fats (predominantly unsaturated ones) in the diet, the composition of different foods must be known (Table 2).

The ideal ratio of different types of fats in a healthy diet is important for maintaining balanced health and reducing the risk of chronic diseases. The recommended Omega-6 to Omega-3 fatty acids ratio is around 4:1 or lower.

Saturated fats should make up less than 10% of total daily caloric intake, focusing on minimizing trans fats as much as possible. Most fat intake should come from unsaturated fats, including monounsaturated and polyunsaturated fats. Total fat should comprise about 20–35% of total daily calories, with a balanced intake of different fat types [62]. Maintaining a moderate total fat intake supports essential functions like hormone production and cellular health while preventing excessive calorie consumption that can lead to weight gain. By focusing on a balanced intake of healthy fats and minimizing the consumption of unhealthy fats, individuals can support overall health and reduce the risk of chronic diseases.

The Western diet is characterized by a high intake of unhealthy fats like saturated and trans fats, which are prevalent in red, processed, ultra-processed meats, dairy products, and processed foods. This diet typically features an imbalance between Omega-6 and Omega-3 fatty acids, with excess Omega-6s from vegetable oils and a deficiency of Omega-3s from sources like fatty fish and nuts. Additionally, the Western diet often lacks sufficient dietary fiber, contributing to various health issues. This pattern of fat consumption is associated with an increased risk of chronic diseases, including heart disease, obesity, and type 2 diabetes. The Western diet is most commonly found in developed countries, particularly North America, Western Europe, and parts of Oceania, such as Australia and New Zealand. It is also becoming increasingly prevalent in urban areas of developing countries as they undergo economic growth and globalization. This diet is typically associated with a lifestyle that includes high consumption of processed and fast foods, convenience meals, and foods rich in fats, sugars, and refined grains. The spread of this dietary pattern is often linked to industrialization, urbanization, and changes in food production and distribution systems, which make processed foods more accessible and affordable [74].

The Mediterranean diet is rooted in the traditional eating habits of countries bordering the Mediterranean Sea, such as Greece, Italy, and Spain. It emphasizes consuming fresh, whole foods, including abundant vegetables, fruits, legumes, nuts, seeds, and whole grains. Olive oil is the primary source of fat, and the diet includes moderate amounts of fish, poultry, and dairy, with red meat and sweets being consumed sparingly. Rich in antioxidants and healthy fats—especially Omega-3s from fish and monounsaturated fats from olive oil—this extensively studied diet promotes heart health, supports longevity, and lowers inflammation levels [75]. The co-occurrence of PUFAs and polyphenols in plant-based foods highlights the sophistication of nature’s design. These bioactives are not randomly distributed but are present in foods humans have historically consumed together. From traditional diets like the Mediterranean, which pairs olive oil (PUFAs and polyphenols) with vegetables and legumes, to Asian cuisines combining sesame seeds with turmeric, cultural practices have long harnessed this natural synergy.

The Paleolithic diet, also known as the “Paleo” or “Caveman” diet, is based on the presumed dietary patterns of our hunter-gatherer ancestors during the Paleolithic era. It focuses on whole, unprocessed foods that could be hunted or gathered, such as lean meats, fish, fruits, vegetables, nuts, and seeds. The diet excludes grains, legumes, dairy products, refined sugars, and processed foods, based on the idea that these foods were not part of early human diets and may contribute to modern health issues. Advocates of the Paleo diet claim it promotes weight loss, improved digestion, and better overall health by aligning with human evolutionary nutrition [76].

The Okinawan diet originates from the Okinawa region of Japan, which is known for having one of the highest concentrations of centenarians globally. This diet is primarily plant-based, heavily relying on vegetables, particularly sweet potatoes, a staple food. It includes moderate amounts of rice, tofu, seaweed, and small portions of fish, with very little meat, dairy, and processed foods. The diet is low in calories but high in nutrients, particularly antioxidants and anti-inflammatory compounds, which are believed to contribute to the exceptional longevity and low rates of chronic diseases observed in the Okinawan population [77].

## 4. The Global Impact of High Saturated and Trans-Fat Consumption: Health Risks, Regulatory Gaps, and Strategies for Mitigation

The contemporary diet, particularly in industrialized societies, presents several gaps and challenges concerning the intake of dietary fats. These issues arise from the types and quantities of fats consumed and misconceptions about their health impacts. Modern diets are marked by an overconsumption of Omega-6 fatty acids from processed and fried foods. On the other hand, foods rich in Omega-3 fatty acids are consumed in significantly lower amounts. This imbalance can lead to inflammation and has been associated with a higher risk of chronic health issues such as cardiovascular disease, obesity, and other inflammatory conditions [78].

Despite widespread awareness of their health risks, saturated and trans fats remain prevalent in many diets, particularly in fast food, baked goods, and processed snacks. High intake of these fats is associated with elevated LDL cholesterol levels and an increased risk of heart disease. Although many governments have taken steps to reduce trans fats in the food supply, they are still present in some products, especially in countries with less stringent regulations.

While unhealthy fats are overconsumed, there is often a deficiency in the intake of healthy fats, such as monounsaturated and polyunsaturated fats. These fats, found in foods like avocados, nuts, seeds, and olive oil, are beneficial for heart health, improving lipid profiles, and reducing inflammation. The challenge is that many individuals either do not consume these fats sufficiently or replace them with low-fat products that often contain added sugars and other unhealthy ingredients.

There is widespread confusion and misinformation among the public regarding dietary fats. For decades, fats were broadly vilified, leading to the proliferation of low-fat and fat-free products high in refined carbohydrates and sugars. This shift has contributed to rising rates of obesity and metabolic syndrome. The challenge now is to re-educate the public about the benefits of healthy fats and the importance of fat quality rather than merely reducing fat intake [79].

Public health guidelines have struggled to keep pace with evolving research on dietary fats. Earlier guidelines emphasized reducing total fat intake without sufficiently distinguishing between different types of fats. While more recent guidelines have made these distinctions clearer, there is still a lag in public understanding and behavior. Moreover, the food industry’s response to these guidelines has often been to market low-fat products that are not necessarily healthier, contributing to further confusion.

Access to healthy dietary fats is not uniform across all populations. Socioeconomic factors play a significant role in dietary choices, with healthier fat sources like fish, nuts, and avocados often being more expensive and less accessible to lower-income groups. This disparity contributes to differences in diet quality and health outcomes between socioeconomic groups, highlighting the need for policies that make healthy fats more affordable and accessible [64,65].

The production of certain dietary fats, particularly those derived from animal sources or palm oil, raises environmental and ethical concerns. The challenge here is to balance the need for healthy dietary fats with sustainable and ethical food production practices. Promoting plant-based sources of fats and supporting sustainable agriculture are potential strategies to address these concerns, but they require significant shifts in consumer behavior and agricultural practices.

Addressing these gaps and challenges requires a multifaceted approach involving updated public health guidelines, better consumer education, and policies that promote access to healthy fats while discouraging the consumption of unhealthy ones. Additionally, there needs to be a continued emphasis on the quality of fats consumed, rather than simply focusing on quantity, to improve overall health outcomes. The unequal access to healthy dietary fats is a significant public health challenge that disproportionately affects lower-income populations, contributing to disparities in diet quality and health outcomes. Comprehensive policy interventions are necessary to bridge this gap, focusing on making healthy fats more affordable and accessible. Addressing these socioeconomic disparities can promote better health outcomes for all populations, reducing the burden of diet-related diseases and improving overall public health [78,80].

The consumption of trans fats represents one of the most critical challenges in contemporary diets, with significant implications for public health. Trans fats, mainly industrially produced trans fatty acids (iTFAs), are formed during the partial hydrogenation of vegetable oils—a process that converts liquid oils into semi-solid fats. These fats are widely used in the food industry due to their stability, long shelf life, and cost-effectiveness, especially in products like margarine, baked goods, snack foods, and fried items.

Trans fats markedly increase low-density lipoprotein (LDL) cholesterol levels while reducing high-density lipoprotein (HDL) cholesterol. These lipid profile alterations caused by trans fats are closely associated with a heightened risk of major cardiovascular events, particularly myocardial infarction.

Trans fats contribute to inflammation and impair endothelial function, which are critical factors in the development of cardiovascular diseases. Chronic inflammation, in particular, is a known contributor to plaque formation in arteries. Emerging evidence suggests that trans fats may impair insulin sensitivity, increasing the risk of developing type 2 diabetes. Insulin resistance is a hallmark of this condition, where cells become less responsive to insulin, leading to elevated blood glucose levels. Some studies indicate that trans fats may negatively affect pancreatic function, further exacerbating the risk of type 2 diabetes by impairing insulin production. Although the relationship between trans fats intake and obesity is complex, some research indicates that trans fats may promote abdominal fat accumulation, a type of fat distribution associated with higher metabolic risks. Trans fats are linked to metabolic syndrome—a cluster of conditions that includes high blood pressure, high blood sugar, excess body fat around the waist, and abnormal cholesterol levels. This syndrome increases the risk of heart disease, stroke, and diabetes. Recent research suggests that trans fats may contribute to neuro-inflammation, a key factor in the development of cognitive decline and dementia, including Alzheimer’s disease. The exact mechanisms are still under investigation, but the association between high trans fats intake and impaired cognitive function is becoming more evident [81,82].

Despite the well-documented health risks, several challenges persist in reducing trans fats intake on a global scale:

- While many high-income countries have implemented regulations to limit or ban the use of iTFAs in food products, such rules are not uniformly enforced globally. In low- and middle-income countries, where regulatory frameworks may weaken, trans fats remain prevalent in many food products.
- The food industry’s reliance on trans fats for their cost-effectiveness and desirable properties (e.g., texture, taste, and shelf stability) has slowed the transition to healthier alternatives. While some companies have voluntarily reduced or eliminated trans fats, others continue to use them, particularly in markets with less stringent regulations.
- Many consumers are still unaware of trans fats in their diets and the associated health risks. This lack of awareness is compounded by confusing labeling practices, where trans fats may not be indicated on food labels, especially in countries with less rigorous labeling laws.
- Products labeled as “trans fats-free” can still contain small amounts of trans fats (up to 0.5 g per serving), adding up to multiple servings. This loophole in labeling regulations can mislead consumers into believing they are avoiding trans fats when they are not.
- Healthier alternatives to trans fats, such as fully hydrogenated or tropical oils like palm and coconut, can be more expensive. This price difference is a significant barrier in low-income communities, where cheaper, trans-fat-laden products are often more accessible.
- In some cultures, traditional or popular foods high in trans fats are deeply ingrained in the diet, making it difficult to change consumption patterns without significant public health interventions.
- To mitigate the health risks associated with trans fats, a multifaceted approach is necessary:
- Strengthening and harmonizing global regulations to reduce or eliminate trans fats from the food supply is essential. It includes setting strict limits on trans fats in food products and effectively enforcing them.
- Providing support to SMEs to reformulate products without trans fats can help them transition to healthier alternatives without facing economic disadvantages.
- Public health campaigns should focus on raising awareness about the dangers of trans fats and promoting healthier dietary fats. Clear labeling and consumer education are critical components of these campaigns.
- Engaging communities, particularly those at higher risk due to socioeconomic factors, can help tailor culturally appropriate and effective interventions in reducing trans-fat consumption.
- Continued research into healthier and cost-effective alternatives to trans fats is very important. Innovations in food technology can help create products that meet consumer preferences without the health risks associated with trans fats.
- Longitudinal studies that track the health outcomes of reduced trans-fat consumption are needed to reinforce public health policies and guidelines.

The intake of trans fats remains a significant public health concern due to their strong association with cardiovascular disease, type 2 diabetes, obesity, and other severe health conditions. Addressing this issue requires regulatory action, public education, and ongoing research to develop healthier alternatives and ensure all populations can access safer, nutritious food options. The ultimate goal is to eliminate trans fats from the global food supply and reduce the burden of diet-related chronic diseases.

Regions with very unhealthy dietary fat consumption are often those where processed foods, fast foods, and industrially produced foods are prevalent. These regions tend to have higher intakes of trans and saturated fats and lower intakes of healthy fats like Omega-3 fatty acids.

The United States and Canada face a significant consumption of ultra-processed fast foods. The frequent and excessive intake of these foods is undoubtedly a major contributing factor to the high prevalence of obesity, diabetes, and cardiovascular diseases in these regions. The widespread availability of inexpensive, calorie-dense foods and a fast-paced lifestyle contribute to poor dietary choices, often prioritizing convenience over nutritional quality [83,84].

While there has been some progress in reducing trans fats through regulation, diets in many Western European countries still contain high levels of saturated fats, especially from dairy products, meats, and processed foods. Countries in Eastern Europe, such as Hungary, Poland, and Ukraine, have some of the highest rates of cardiovascular disease globally. Diets in these regions are often high in saturated fats and trans fats due to consuming processed foods, fried foods, and low-quality cooking oils [85].

In recent decades, many countries in the Middle East and North Africa have experienced a dietary shift from traditional diets rich in vegetables, legumes, and fish to more Westernized diets high in saturated and trans fats. This shift is driven by urbanization, economic growth, and increased availability of processed foods. The result has been a rising prevalence of obesity, diabetes, and cardiovascular diseases in these regions, often exacerbated by a sedentary lifestyle and limited access to preventive healthcare [86].

In South Asia, particularly in India, Pakistan, and Bangladesh, partially hydrogenated oils (such as vanaspati ghee) are commonly used in cooking and food preparation. These oils are a significant source of trans fats. High consumption of these unhealthy fats contributes to the region’s growing burden of heart disease, diabetes, and other chronic conditions [87,88].

Latin American countries like Mexico, Brazil, and Argentina have seen a rise in the consumption of processed foods, sugary beverages, and fast foods, leading to increased intake of unhealthy fats. Mexico, in particular, has one of the highest obesity rates in the world, driven by diets high in unhealthy fats, sugars, and refined carbohydrates. Diet-related chronic diseases are also high in this region [89].

In rapidly urbanizing areas of Sub-Saharan Africa, traditional diets are being replaced by diets high in processed foods, refined oils, and fast foods, leading to increased consumption of unhealthy fats. Many countries in Sub-Saharan Africa face a dual burden of malnutrition, where undernutrition exists alongside rising rates of obesity and non-communicable diseases driven by poor dietary quality [90].

In Southeast Asia, the widespread use of palm oil, which is high in saturated fats, contributes to unhealthy fat consumption. Palm oil is a cooking and food-processing staple in Indonesia, Malaysia, and the Philippines. The growing influence of Western fast food chains and processed foods has further increased the intake of unhealthy fats, contributing to rising obesity and non-communicable diseases [91].

Unhealthy fat consumption is a global issue, but it is particularly pronounced in regions with high consumption of processed foods, fast foods, and hydrogenated oils. Addressing this issue requires targeted public health interventions, improved food labeling, and efforts to promote healthier dietary patterns that prioritize the consumption of unsaturated fats from sources like fish, nuts, seeds, and plant oils.

## 5. Biotransformation and Biological Impact of Polyunsaturated Fatty Acids in Human Physiology

**Figure 3:**
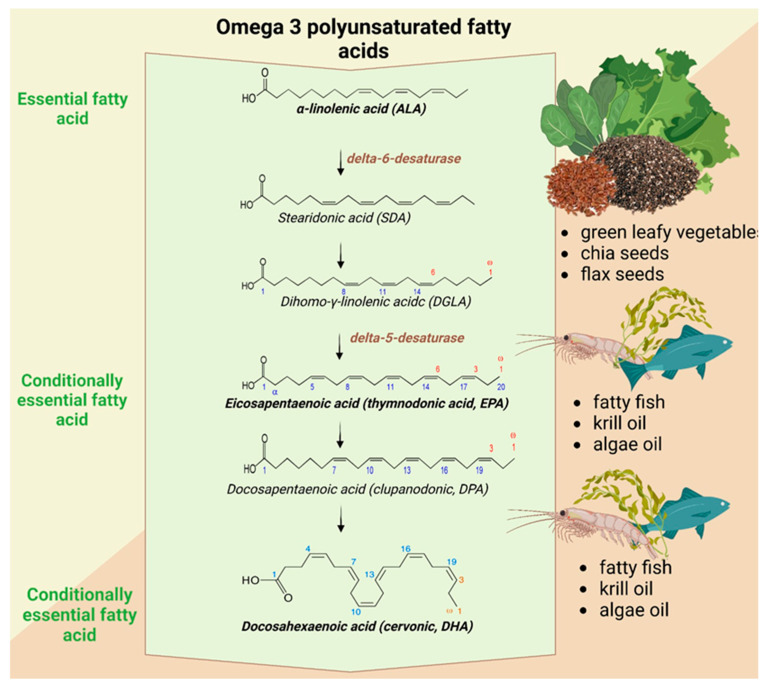
The stages of biotransformation of ALA in the human body. Created with BioRender.com (accessed on 15 October 2024).

The biotransformation of essential fatty acids into long-chain polyunsaturated fatty acids involves a complex interplay of desaturation and elongation steps, mediated by key enzymes such as delta-6 and delta-5 desaturases and elongase enzymes. These transformations enable the production of bioactive lipids that play a pivotal role in human health, particularly in regulating inflammation and immune responses. After ingestion, ALA undergoes a series of metabolic transformations that take place mainly at the hepatic level and which convert it into long-chain PUFAs, such as EPA and DHA, with participation of various enzymes that facilitate desaturation and elongation reactions (Figure 3) [92,93]. However, only a small fraction of dietary ALA is efficiently converted into these biologically active derivatives, with the majority remaining as ALA. The first critical step in ALA metabolism is its conversion to stearidonic acid (SDA) through the action of delta-6 desaturase (FADS2). This enzyme inserts a double bond into the carbon chain of ALA, creating a more unsaturated fatty acid. Delta-6 desaturase is considered a rate-limiting enzyme in the entire pathway, and its activity can vary depending on individual genetics, age, and overall health status. After the formation of SDA, the molecule undergoes elongation by elongase enzymes (mainly ELOVL5). These enzymes extend the carbon chain by adding two additional carbon atoms, producing ETA. The next step involves the action of delta-5 desaturase (FADS1), which introduces another double bond, converting ETA into EPA [93]. EPA is a key Omega-3 fatty acid known for its anti-inflammatory properties and role in cardiovascular and immune health. EPA can be further elongated and desaturated to form DHA. The conversion of EPA to DHA involves additional elongation steps and the action of delta-6 desaturase, although the efficiency of this process in humans is relatively low. DHA is an essential component of cell membranes, particularly in the brain, retina, and nervous system, where it supports cognitive function, vision, and neurological health [94].

Biochemically, ALA can be converted into DHA and EPA; however, this biotransformation process is influenced by various factors [40,93,95]:

Genetic variation: differences in the efficiency of delta-5 and delta-6 desaturase enzymes, which are important for converting ALA to EPA and DHA, may arise due to genetic polymorphisms in the FADS1 and FADS2 genes. Thus, these variations can significantly reduce the conversion rate.

The antagonism between Omega-6 and Omega-3 pathways: An excessive intake of Omega-6 fatty acids can further inhibit the conversion of ALA into EPA and DHA. It occurs because Omega-6 and Omega-3 fatty acids share the same metabolic enzymes, including desaturases and elongases, leading to competition within these pathways.

Dietary and health factors: the efficiency of ALA biotransformation is also influenced by dietary factors such as overall fat intake, age, sex, and metabolic health. For instance, conditions like insulin resistance or obesity may impair the body’s ability to efficiently convert ALA into its long-chain metabolites. Although ALA is a valuable dietary source of Omega-3s, obtaining EPA and DHA directly from marine sources may be essential to achieve optimal health benefits.

The biotransformation of ALA into EPA and DHA is a critical metabolic process that enables the production of biologically active compounds. EPA is a precursor to eicosanoids, such as prostaglandins, thromboxanes, and leukotrienes. Prostaglandins control inflammation and healing, thromboxanes regulate blood clot formation, and leukotrienes influence immune responses. By modulating these pathways, EPA and DHA maintain physiological balance and prevent excessive inflammation or clotting, key factors in many chronic diseases. EPA reduces blood triglyceride levels, improves endothelial function, and prevents excessive platelet aggregation, all of which protect against cardiovascular diseases and help modulate the body’s inflammatory responses, making it beneficial for individuals with chronic inflammatory conditions such as arthritis or cardiovascular diseases [31].

DHA is essential for maintaining the structural integrity of cell membranes, particularly in neurons and photoreceptor cells in the retina. It is critical for brain development and function, and its deficiency has been linked to cognitive decline and neurodegenerative diseases. DHA predominates in the brain, which plays a significant role in synaptic plasticity, signaling, and cognitive performance. Optimal levels of DHA have been associated with enhanced cognitive abilities, including improved memory and learning processes. Additionally, DHA influences mood regulation, contributing to overall mental well-being. DHA is a key structural component of the retina, contributing to optimal visual function. Deficiencies in DHA can lead to visual impairment and other eye-related issues [96].

Excessive intake of DHA and EPA may lead to several adverse effects despite their known health benefits at moderate levels. High doses of DHA and EPA can interfere with normal blood clotting processes, potentially increasing the risk of bleeding or bruising due to their blood-thinning properties. In some cases, excessive Omega-3 intake has been linked to an increased susceptibility to immune system suppression, which could impair the body’s ability to fight infections. Furthermore, high levels of these fatty acids may disrupt the balance of fatty acids in the body, possibly leading to an imbalance in lipid metabolism, altering the function of cell membranes, and affecting cellular signaling pathways. While the anti-inflammatory properties of DHA and EPA are beneficial, excessive intake may paradoxically lead to undesirable immune responses or exacerbate inflammation in certain individuals. Therefore, while Omega-3 fatty acids are essential for health, their intake should be carefully monitored to avoid potential negative effects of overconsumption [31,96].

Since the human body cannot synthesize LA, it must be obtained through dietary sources such as vegetable oils, nuts, and seeds. Once consumed, LA undergoes a series of biotransformations that produce key metabolites involved in inflammation, immunity, and cell membrane integrity. The metabolic conversion of LA into biologically active molecules involves specific enzymatic reactions, and the products of these conversions serve vital functions in human health.

**Figure 4:**
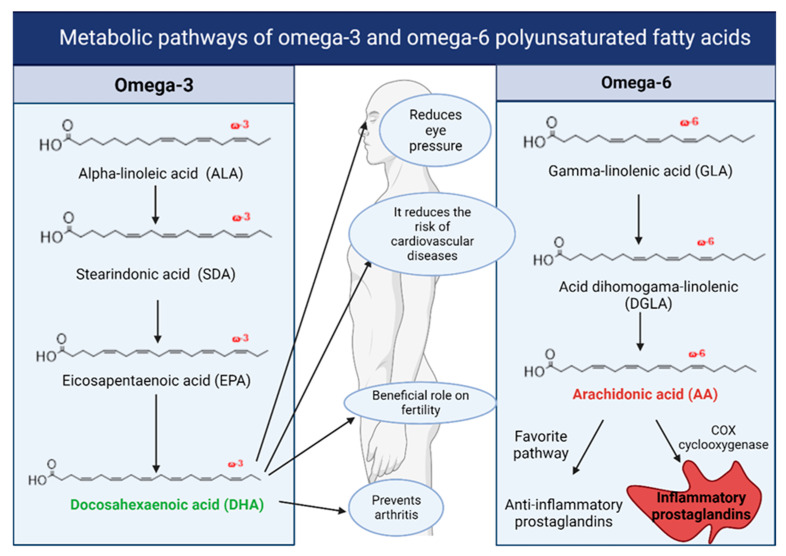
Biotransformation of essential fatty acids in the human body. Created with BioRender.com (accessed on 15 October 2024).

Similar to ALA, LA is metabolized through a series of desaturation and elongation steps. These reactions primarily occur in the liver, where enzymes such as desaturases and elongases facilitate the conversion of LA into long-chain polyunsaturated fatty acids (Figure 4). The initial stage of LA metabolism is catalyzed by delta-6 desaturase (FADS2), which adds a double bond between the 6th and 7th carbon atoms of the fatty acid chain. This reaction converts LA into GLA, an Omega-6 fatty acid precursor, producing bioactive lipids. After GLA is formed, it undergoes elongation by elongase enzymes, primarily ELOVL5. This reaction adds two carbon atoms to the chain, forming di-homo-gamma-linolenic acid (DGLA). The next step involves the conversion of DGLA into AA through the action of delta-5 desaturase (FADS1). Arachidonic acid is a major LA metabolism product and is a critical precursor for eicosanoid synthesis (prostaglandins, thromboxanes, leukotrienes) [40].

Several factors influence the efficiency and balance of LA biotransformation into its downstream products, including genetic variations, dietary patterns, and competition with Omega-3 fatty acid metabolism [40,93,95]:

Genetic variations: polymorphisms in the genes encoding delta-5 and delta-6 desaturases (FADS1 and FADS2) can affect an individual’s ability to convert LA into its metabolites efficiently. Variations in these enzymes can decrease the conversion rate, resulting in lower levels of GLA, DGLA, and AA, which may affect an individual’s risk of developing inflammatory conditions and cardiovascular diseases.

A balanced ratio between Omega-3 and Omega-6 fatty acids, achieved and maintained through a healthy and diverse dietary pattern, improves overall health, supports well-being, and enhances quality of life by preventing or alleviating chronic diseases. LA (Omega-6) and ALA (Omega-3) compete for the same desaturase and elongase enzymes in their metabolic pathways. A diet rich in Omega-6 but low in Omega-3 can shift metabolism toward increased production of pro-inflammatory eicosanoids from AA, reducing the anti-inflammatory effects of EPA and DHA from the Omega-3 pathway.

Overall dietary and health factors: nutritional status, including the intake of micronutrients such as zinc and magnesium, which are cofactors for desaturase enzymes, can impact the biotransformation of LA. Additionally, metabolic health factors such as insulin resistance, obesity, and aging may impair the body’s ability to convert LA into its beneficial metabolites.

The metabolites of LA, particularly GLA, DGLA, and AA, serve important biological functions, especially in regulating inflammatory processes and maintaining cell membrane structure. GLA is an important intermediate in LA metabolism. Although its direct biological role is relatively minor compared to its metabolites, GLA can exert anti-inflammatory effects by producing DGLA-derived molecules. GLA is vital in preserving skin integrity by supporting its structure and barrier function. Deficiencies in GLA are commonly linked to skin conditions like eczema and dermatitis [97].

While Omega-6 fatty acids are often associated with pro-inflammatory effects, GLA has anti-inflammatory properties, particularly when converted to DGLA.

DGLA is a precursor to several anti-inflammatory molecules, including 15-hydroxyeicosatrienoic acid (15-HETrE) and prostaglandin E1 (PGE1). Unlike arachidonic acid, which often leads to the production of pro-inflammatory eicosanoids, DGLA tends to produce molecules that help resolve inflammation and maintain cellular balance. DGLA-derived products, particularly PGE1, have immune-modulating effects, helping to control the body’s inflammatory response. DGLA is beneficial for conditions like rheumatoid arthritis and other inflammatory diseases. PGE1, a product of DGLA, supports cardiovascular health by promoting vasodilation, improving blood flow, reducing the risk of blood clots, and lowering blood pressure [97].

AA is a precursor for synthesizing prostaglandins, thromboxanes, and leukotrienes. These molecules regulate inflammation, immune responses, and vascular functions. While AA is vital for cellular signaling and immune response, the eicosanoids derived from AA, such as prostaglandin E2 (PGE2) and leukotriene B4 (LTB4), are often associated with promoting inflammation. These compounds are important in mediating acute inflammatory responses but can contribute to chronic inflammation if not properly regulated. AA-derived prostaglandins and leukotrienes are essential in tissue repair and defending the body against infections. AA is released from the cell membrane during injury or infection to promote localized inflammation and healing processes [98].

The metabolites of essential fatty acids, particularly AA and EPA, serve as precursors for producing eicosanoids. These compounds regulate inflammation, immune function, and other cellular processes. A balance between Omega-6-derived and Omega-3-derived eicosanoids is vital for maintaining homeostasis. A dietary imbalance favoring Omega-6 PUFAs over Omega-3 PUFAs can promote a pro-inflammatory state, contributing to various chronic diseases. The balance between pro-inflammatory and anti-inflammatory products of LA metabolism is key to maintaining overall health, and factors such as genetic variations, dietary intake, and the Omega-6 to Omega-3 ratio can influence the efficiency of this metabolic pathway. While LA and its derivatives play essential roles, it is important to maintain a balanced intake of fatty acids to optimize their beneficial effects and minimize the risk of chronic inflammation [99].

## 6. Key Functions of Essential Fatty Acids

**Figure 5:**
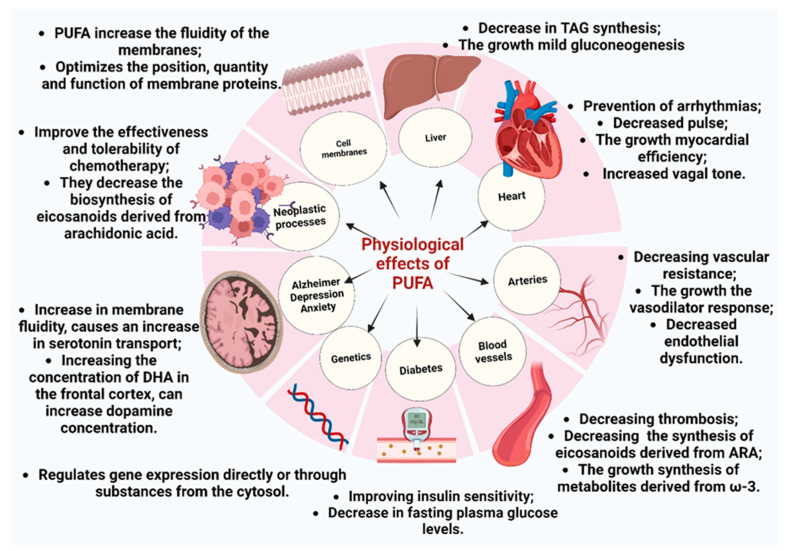
Physiological effects of PUFAs. Created with BioRender.com (accessed on 15 October 2024).

Essential fatty acids and their metabolites are vital in cell membrane integrity, inflammation regulation, brain function, and cardiovascular health (Figure 5). A deficiency in these fatty acids can lead to various physiological disruptions, with consequences that affect multiple organ systems and overall well-being [100].

Numerous clinical studies have explored the benefits of PUFAs in various health domains. Table 3 summarizes key findings from significant studies.

### 6.1. Cell Membrane Integrity

EFAs are essential to maintaining cell membranes’ structural and functional integrity, primarily composed of lipid bilayers. The presence of EFAs contributes to the fluidity of these membranes by preventing the fatty acid chains from becoming too rigid or tightly packed. This membrane fluidity is important for properly functioning embedded proteins, such as receptors and ion channels, which play key roles in cell signaling. EFAs allow membranes to remain flexible, facilitating dynamic movement and interaction of signaling molecules. Moreover, EFAs are precursors to signaling molecules like eicosanoids and docosanoids, which regulate inflammation, immune responses, and other cellular functions. Without sufficient EFAs, membranes become more rigid and less capable of supporting efficient signal transduction, impairing cellular communication and function across various physiological processes [110].

Essential fatty acids are key precursors for bioactive lipid mediators, such as prostaglandins, leukotrienes, and resolvins, which regulate inflammatory and immune responses. EFAs, particularly Omega-6 (arachidonic acid) and Omega-3 (eicosapentaenoic acid), are converted into these molecules through enzymatic processes like desaturation and elongation. Prostaglandins, produced from AA, are involved in acute inflammation by promoting vasodilation and recruiting immune cells to sites of injury or infection. Leukotrienes, derived from AA, are also implicated in chronic inflammatory processes, particularly when their production is excessively stimulated [111].

### 6.2. Brain Function

**Figure 6:**
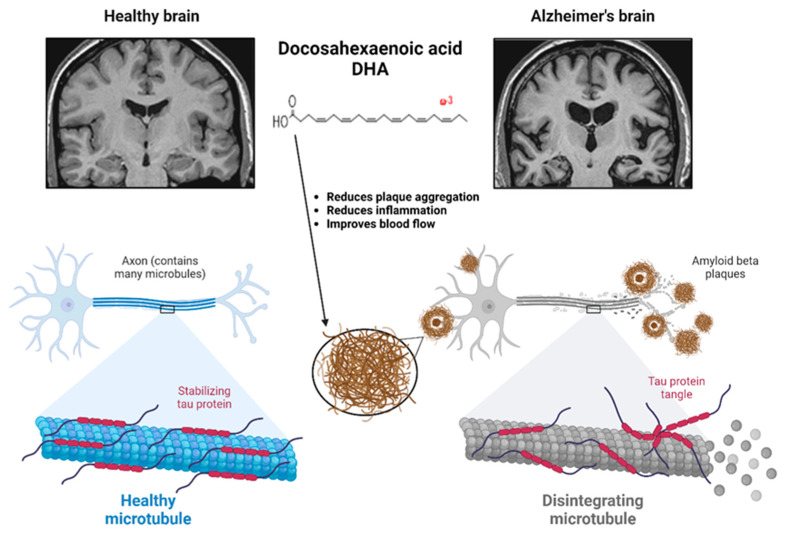
DHA and healthy brain. Created with BioRender.com (accessed on 15 October 2024).

Omega-3 fatty acids are vital for brain development. DHA is the brain’s most abundant Omega-3 fatty acid, accounting for a significant portion of the phospholipid content in neuronal cell membranes (Figure 6). Its presence is vital for maintaining the fluidity and flexibility of these membranes, which directly impacts the function of membrane-bound proteins such as neurotransmitter receptors, ion channels, and transporters necessary for synaptic transmission and plasticity [112].

DHA’s fluidizing effect on cell membranes enhances the capacity for signal transduction, facilitating efficient communication between neurons. It is essential for higher-order cognitive processes such as learning, memory consolidation, and problem-solving. During fetal and early childhood brain development, DHA is significant, as it supports the growth of dendrites and synaptic connectivity, which are fundamental for the maturation of the central nervous system. Adequate DHA levels during critical periods of development are necessary to ensure proper neuronal structure and the formation of healthy neural circuits [113].

While present in smaller quantities in the brain than DHA, EPA plays a complementary role in neurological health. It is a precursor to anti-inflammatory eicosanoids, essential for regulating neuroinflammation. Chronic inflammation in the brain can lead to neuronal damage and is associated with neurodegenerative conditions such as Alzheimer’s disease and Parkinson’s disease.

In addition to their structural and anti-inflammatory roles, Omega-3 fatty acids are involved in the modulation of key neurotransmitter systems. DHA influences the production and function of neurotransmitters like acetylcholine, glutamate, and gamma-aminobutyric acid (GABA), which are critical for cognitive functions such as memory, attention, and mood regulation. Meanwhile, EPA is linked to regulating dopamine and serotonin pathways, central to mood stabilization and emotional well-being. It is why deficiencies in Omega-3s, particularly EPA and DHA, have been associated with an increased risk of mood disorders, such as depression and anxiety, as well as cognitive decline and neurodevelopmental issues in children [114,115].

Furthermore, Omega-3s influence the expression of genes related to neurogenesis, neuroplasticity, and synaptic function. Modulating gene expression promotes the repair and growth of neurons, essential for maintaining brain health throughout life, particularly in response to injury or aging.

In summary, Omega-3 fatty acids EPA and DHA are essential for brain function throughout all life stages, from early development to old age. They support the structural integrity of neurons, regulate inflammation, modulate neurotransmitter systems, and influence gene expression, all of which are critical for maintaining cognitive health, mood balance, and the protection of neural tissues against degeneration. Deficiency in these key fatty acids can significantly impair brain development, cognitive function, and overall neurological health.

### 6.3. Cardiovascular Health

Essential fatty acids, particularly Omega-3 fatty acids such as EPA and DHA, are important for regulating blood lipid levels and significantly influencing cardiovascular health. A major way Omega-3s help lower blood lipid levels is by reducing triglyceride concentrations. They enhance the activity of lipoprotein lipase (LPL). This enzyme breaks down triglycerides in lipoproteins into free fatty acids, which tissues can use for energy or stored in fat cells. This enzymatic process helps reduce circulating triglyceride levels, which, when elevated, are a significant risk factor for cardiovascular diseases. Fatty acids influence lipid metabolism by directly modulating gene expression. They activate peroxisome proliferator-activated receptor alpha (PPAR-α), a key factor in controlling fatty acid oxidation and triglyceride breakdown. This activation stimulates the expression of specific genes that facilitate the degradation of lipids. As a result, Omega-3s contribute to a measurable reduction in triglyceride levels within the liver, supporting improved lipid management and metabolic health [94,116].

Omega-3 fatty acids also inhibit the expression of genes such as sterol regulatory element-binding protein 1c (SREBP-1c) and fatty acid synthase (FAS). These genes play a central role in lipogenesis, the metabolic process of synthesizing fatty acids and triglycerides. By downregulating these pathways, Omega-3s help reduce fat accumulation.

In addition to their effects on lipid metabolism, Omega-3s exhibit strong anti-inflammatory properties important for cardiovascular protection. They serve as precursors to bioactive compounds like resolvins and protectins, which help resolve inflammation by reducing the production of pro-inflammatory cytokines, including tumor necrosis factor-alpha (TNF-α) and interleukin-6 (IL-6). Chronic low-grade inflammation is a key factor in the progression of atherosclerosis, marked by plaque buildup in the arteries, which raises the risk of heart attacks and strokes. Omega-3s also influence the expression of cyclooxygenase-2 (COX-2), an enzyme that plays a central role in the inflammatory process. By reducing COX-2 expression, Omega-3s further decrease the production of inflammatory mediators that can exacerbate vascular inflammation [117,118].

Additionally, Omega-3 fatty acids impact endothelial function, which is essential for vascular health. They promote the release of nitric oxide (NO), a potent vasodilator produced by endothelial cells, thereby improving blood flow and helping to lower blood pressure. The balance of lipid levels, anti-inflammatory effects, and improved endothelial function collectively contribute to a reduced risk of cardiovascular events.

In summary, essential fatty acids (EFAs), particularly Omega-3s, help reduce triglyceride levels by activating enzymes like LPL and regulating genes such as PPAR-α and SREBP-1c. They also provide anti-inflammatory benefits by modulating cytokine production and COX-2 expression. These interconnected mechanisms underscore the critical role of Omega-3 fatty acids in supporting cardiovascular health and lowering the risk of heart disease [119].

### 6.4. Cancer Prevention

PUFAs, particularly Omega-3 and Omega-6, play a significant role in cancer biology, influencing the disease’s development and progression by modulating various molecular pathways. EPA and DHA exhibit anti-cancer properties by regulating the expression of genes associated with cell growth, apoptosis, and inflammatory processes. For example, Omega-3 fatty acids can upregulate the expression of PPARs, specifically PPAR-gamma. This receptor facilitates apoptosis in cancer cells while suppressing their proliferation, contributing to its potential anti-cancer effects.

High levels of Omega-3s may impair blood clotting by reducing platelet aggregation, which increases the risk of bleeding and bruising. Additionally, excessive intake can suppress immune function, potentially making the body more vulnerable to infections. Overconsumption of Omega-3s may also disrupt the body’s balance between Omega-6 and Omega-3 fatty acids, potentially leading to imbalances in lipid metabolism and affecting cell membrane fluidity and function. This imbalance could sometimes alter cellular signaling pathways, impacting various physiological processes. Furthermore, while Omega-3s have anti-inflammatory properties, excessive amounts may paradoxically provoke inflammatory responses in certain individuals, particularly those with underlying health conditions. Therefore, maintaining a balanced intake of Omega-3 fatty acids is essential for optimal health, as excessive amounts can lead to unintended adverse effects [120].

In contrast, excess Omega-6 fatty acids, such as arachidonic acid, can activate inflammatory pathways that promote tumorigenesis. It occurs by upregulating enzymes like cyclooxygenase-2 (COX-2) and lipoxygenase (LOX), producing pro-inflammatory eicosanoids, including prostaglandins and leukotrienes. These eicosanoids can support cell survival, angiogenesis, and metastasis, creating a microenvironment that facilitates tumor growth. Additionally, the Omega-3 to Omega-6 ratio influences the activity of transcription factors like nuclear factor kappa B (NF-κB), which is involved in inflammatory responses and has been linked to various cancers. By altering the fatty acid composition in cell membranes, polyunsaturated fatty acids (PUFAs) can modify membrane fluidity and receptor activity, thus impacting signaling pathways that control cell growth and differentiation. Overall, the intricate interplay between PUFAs, gene expression, and enzymatic activity highlights their vital role in modulating neoplastic processes and underscores the importance of dietary fat composition in cancer prevention and therapy [120,121].

### 6.5. Insulin Sensitivity

**Figure 7:**
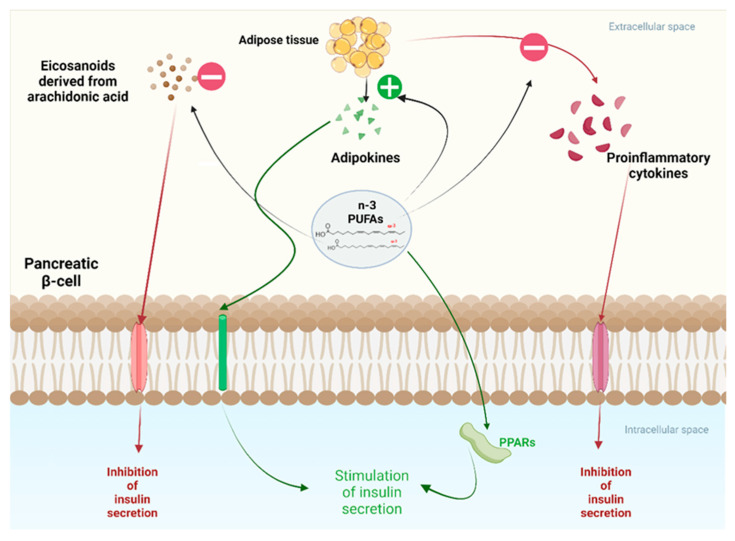
Omega-3 fatty acids and insulin secretion. Created with BioRender.com (accessed on 15 October 2024).

Studies suggest that Omega-3 fatty acids, such as EPA and DHA, enhance insulin sensitivity through multiple mechanisms (Figure 7). A primary pathway includes the activation of PPARs, with a notable focus on PPAR-gamma. This receptor regulates glucose homeostasis by promoting glucose uptake into adipocytes and muscle cells and modulating lipid metabolism. PPAR-gamma activation also facilitates the differentiation of pre-adipocytes into mature adipocytes, which can improve insulin sensitivity by enhancing lipid storage and reducing free fatty acid levels in circulation.

Omega-3 fatty acids possess anti-inflammatory properties that help combat insulin resistance. Chronic inflammation, driven by elevated pro-inflammatory cytokines like TNF-α and IL-6, is a major contributor to its development. Omega-3s inhibit the NF-kB signaling pathway, which controls the expression of these inflammatory cytokines. Omega-3 fatty acids help improve insulin signaling pathways by reducing inflammation, thereby increasing insulin sensitivity [31,122]. Contrary to this, excessive Omega-6 fatty acids, particularly arachidonic acid, may promote insulin resistance. It occurs through mechanisms such as increased production of inflammatory eicosanoids via the COX-2 and lipoxygenase (LOX) pathways. These pro-inflammatory mediators can impair insulin signaling by disrupting the insulin receptor substrate (IRS) pathway, leading to reduced glucose transporter type 4 (GLUT4) translocation to the cell membrane. This impairment decreases glucose uptake in muscle and fatty tissues, contributing to higher blood glucose levels and worsening insulin sensitivity [123].

Notably, Omega-6 fatty acids can lead to the accumulation of visceral fat, a condition closely tied to increased insulin resistance. Visceral fat releases adipokines that impair insulin sensitivity, and an imbalance between Omega-6 and Omega-3 fatty acids creates a pro-inflammatory environment, further contributing to insulin resistance [124].

Additionally, the role of endoplasmic reticulum (ER) stress has been highlighted in the context of insulin resistance. The imbalance in fatty acid composition can induce ER stress, activating the unfolded protein response (UPR) and further impairing insulin signaling. The intricate interplay between fatty acid composition, inflammation, and cellular stress emphasizes the pivotal role of maintaining a balanced intake of PUFAs in preventing and managing insulin resistance and type 2 diabetes. Omega-3 fatty acids enhance insulin sensitivity through PPAR-gamma activation and anti-inflammatory mechanisms. In contrast, excessive Omega-6 fatty acid intake exacerbates insulin resistance via inflammatory pathways, dysregulated adipokine secretion, and the promotion of endoplasmic reticulum (ER) stress [46,124].

### 6.6. Integrity of the Skin Barrier

Omega-6 fatty acids, such as linoleic acid, are essential for maintaining the integrity of the skin barrier by promoting the synthesis of ceramides and other lipids that form the stratum corneum, the outermost layer of the skin. This barrier function is important for preventing transepidermal water loss and protecting against environmental insults. The enzyme Δ6-desaturase is critical for converting linoleic acid into GLA, a precursor for anti-inflammatory prostaglandins. These prostaglandins regulate skin inflammation and facilitate wound healing [125].

Conversely, Omega-3 fatty acids, especially EPA and DHA, promote skin health by regulating inflammatory pathways and enhancing the skin’s ability to retain moisture. EPA helps reduce the production of pro-inflammatory cytokines such as interleukin-1 (IL-1) and tumor necrosis factor-alpha (TNF-α) by inhibiting the NF-κB signaling pathway. By reducing inflammation, Omega-3s help alleviate conditions such as acne, psoriasis, and atopic dermatitis, which are characterized by excessive inflammatory responses. Additionally, DHA promotes skin hydration by modulating the expression of aquaporins, which are water channel proteins essential for maintaining cellular moisture levels [23,31].

Furthermore, PUFAs can also impact skin aging by influencing gene expression related to collagen synthesis and oxidative stress. For instance, Omega-3 fatty acids enhance the expression of matrix metalloproteinases (MMPs) involved in tissue remodeling and repair while simultaneously regulating genes that produce antioxidants that combat oxidative damage. This dual action helps maintain skin elasticity and resilience, reducing the appearance of fine lines and wrinkles [126].

In conclusion, the skin’s health is intricately linked to the balance of PUFAs, with Omega-6 fatty acids supporting barrier function and inflammation resolution. In contrast, Omega-3 fatty acids enhance hydration and combat oxidative stress. The interplay between these fatty acids and various enzymes and genes underscores their essential role in promoting overall skin health and mitigating the effects of skin disorders and aging.

## 7. Consequences of EFA Deficiency

Deficiency in essential fatty acids can have widespread consequences, affecting everything from skin health and cognitive function to cardiovascular and immune system regulation. The severity and type of consequences depend on the deficiency (Omega-6 or Omega-3) and the duration and extent of the insufficiency. Maintaining a balanced intake of Omega-6 and Omega-3 fatty acids is important for supporting optimal physiological functions and preventing chronic diseases linked to inflammation, metabolic imbalances, and neurological issues. Dietary habits, health status, and genetic factors influence the body’s essential fatty acid levels, underscoring the significance of sufficient and balanced nutrition [46,113].

Several factors can contribute to the development of EFA deficiency:

Dietary imbalance: a diet high in processed foods rich in Omega-6 fatty acids and low in Omega-3 sources can lead to an imbalance, promoting inflammatory processes and depriving the body of adequate Omega-3s for optimal function.

Genetic factors: Some individuals have genetic variations that reduce the activity of enzymes, such as delta-6 desaturase, which is responsible for converting short-chain EFAs like ALA into longer-chain PUFAs (EPA and DHA). It can increase the need to directly intake longer-chain Omega-3 fatty acids from sources like fish oil. Certain medical conditions that impair fat absorption, such as cystic fibrosis, celiac disease, or chronic pancreatitis, can also lead to deficiencies in EFAs.

Excessive consumption of PUFAs, particularly Omega-6 fatty acids, can harm health. The high Omega-6 to Omega-3 ratio commonly found in Western diets can trigger an overproduction of pro-inflammatory eicosanoids, such as prostaglandins and leukotrienes, derived from arachidonic acid, an Omega-6 fatty acid. It occurs through the activity of enzymes like cyclooxygenase (COX) and lipoxygenase (LOX), which convert arachidonic acid into inflammatory mediators. Elevated levels of these eicosanoids can worsen inflammatory conditions like arthritis, asthma, and cardiovascular diseases, leading to tissue damage and disease progression [23,58,127].

Excessive Omega-6 fatty acids can disrupt Omega-3 metabolism by competing for shared enzymes, including Δ6-desaturase and Δ5-desaturase. This competition limits the conversion of Omega-3 fatty acids, such as ALA, into their active forms, EPA, and DHA, reducing their anti-inflammatory benefits. The resulting imbalance in the fatty acid composition within cell membranes can alter membrane fluidity and disrupt the function of membrane-bound proteins, including receptors and signaling pathways essential for cellular communication.

Additionally, excess PUFAs can lead to oxidative stress, particularly when Omega-6 fatty acids are excessively oxidized during cooking or food processing. This oxidative degradation produces harmful free radicals and lipid peroxidation products, which can damage cellular structures, including lipids, proteins, and DNA. The accumulation of oxidative damage can trigger cellular stress responses and activate signaling pathways involving transcription factors such as nuclear factor kappa B (NF-κB), further exacerbating inflammation and increasing the risk of chronic diseases [128].

Essential fatty acids are essential for maintaining the skin’s barrier function and hydration; a deficiency in these vital nutrients can lead to various dermatological issues, such as xerosis, poor wound healing, and hair loss. Xerosis, characterized by dry, scaly skin, occurs when the skin barrier is compromised, increasing transepidermal water loss. This deficiency reduces the synthesis of ceramides and other lipids essential for the integrity of the stratum corneum, mediated by enzymes such as Δ6-desaturase, which converts linoleic acid into gamma-linolenic acid, crucial for producing anti-inflammatory mediators. As the barrier function diminishes, the skin becomes more susceptible to irritation, itching, and secondary infections due to impaired immune responses in the epidermis.

Moreover, EFAs are integral to the skin’s wound-healing processes. They promote the synthesis of eicosanoids, including prostaglandins and leukotrienes, through the action of cyclooxygenase (COX) enzymes. These eicosanoids are vital for regulating inflammation and initiating the healing cascade by promoting angiogenesis and fibroblast activity. A deficiency in EFAs can disrupt these processes, leading to slower wound healing and an increased risk of complications following skin injuries [129,130,131].

EFAs contribute significantly to cells’ structural and functional integrity, particularly in the context of hair and scalp health. These bioactive lipids are recognized for supporting hair follicle function by enhancing local blood flow and ensuring effective nutrient delivery to the scalp, thereby promoting overall follicular health and vitality.

A deficiency in EFAs can lead to alopecia (hair loss) and brittle hair due to the compromised integrity of hair follicles and the scalp’s cellular environment. It can involve alterations in gene expression related to keratin synthesis and cell proliferation, negatively impacting hair growth and resilience [126].

In conclusion, essential fatty acids maintain skin hydration, heal wounds, and promote hair health. Their deficiency can lead to xerosis, impaired healing, and hair loss due to lipid metabolism, inflammatory response, and cellular integrity disruptions, highlighting the importance of adequate EFA intake for overall skin and hair health.

Essential fatty acids, particularly Omega-3s, play a fundamental role in the growth and development of infants and children. These nutrients are of utmost importance for proper brain and nervous system development, and their deficiency can result in stunted growth and developmental delays. The need for Omega-3 fatty acids, especially DHA, is significantly elevated during infancy and early childhood. DHA is vital for forming and maturing neuronal and glial cells, essential for the brain’s structural and functional development. The rapid brain growth during these stages relies on sufficient DHA levels to support synaptogenesis, myelination, and overall neural connectivity, highlighting its importance in early-life nutrition. A deficiency in DHA can impair the expression of critical neurodevelopmental genes, such as those regulating neurogenesis, neuronal differentiation, and synaptogenesis, potentially leading to long-lasting cognitive deficits [132,133].

Furthermore, the importance of DHA extends to visual development, as it is a major structural component of the retina. Its presence in photoreceptor cells is essential for optimal visual function, including converting light into electrical signals. Deficiencies during crucial developmental windows—such as pregnancy or early childhood—can impair retinal development, affecting visual acuity and potentially leading to long-term visual impairments. The enzyme Δ5-desaturase, essential for converting ALA into DHA, is sensitive to insufficient ALA intake, potentially reducing DHA synthesis and worsening deficiencies [134].

Omega-3 fatty acids, particularly DHA and EPA, support cognitive function and emotional health.

A deficiency in these essential fatty acids has been linked to cognitive decline, memory loss, and difficulties with concentration. DHA is a major structural component of neuronal membranes, contributing to membrane fluidity and facilitating synaptic signaling. Low levels of DHA can impair the function of key neurotransmitter systems, particularly serotonin and dopamine, which are critical for mood regulation. The enzyme phospholipase A2, which releases fatty acids from membrane phospholipids, can be negatively impacted by inadequate Omega-3 levels, leading to altered membrane composition and disrupted neurotransmission. This disruption is associated with increased inflammation in the brain, as EPA and DHA possess anti-inflammatory properties that help modulate the production of pro-inflammatory cytokines such as interleukin-1 (IL-1) and tumor necrosis factor-alpha (TNF-α). Chronic inflammation in the central nervous system has been linked to mood disorders like depression and anxiety, creating a feedback loop where inflammation further exacerbates cognitive decline and emotional instability [113,135].

Additionally, essential fatty acids are important for peripheral nerve health, and their deficiency can contribute to peripheral neuropathy, a condition characterized by symptoms such as numbness, tingling, and muscle weakness, particularly in the extremities. Omega-3s help maintain the integrity of myelin, the protective sheath surrounding nerve fibers, promoting efficient nerve signal transmission. Inadequate levels of Omega-3s can impair the synthesis of sphingolipids and other lipids crucial for myelin formation, resulting in demyelination and nerve dysfunction. This dysfunction can lead to the activation of signaling pathways involving inflammatory mediators, further exacerbating nerve damage.

Thus, a deficiency in Omega-3 fatty acids, particularly DHA and EPA, is linked to cognitive decline, mood disorders, and peripheral neuropathy. The underlying mechanisms of these effects include disrupted neurotransmitter function, heightened inflammation, and compromised nerve health due to changes in membrane composition and myelin integrity. It is crucial to ensure an adequate intake of Omega-3 fatty acids to support cognitive function, emotional well-being, and nerve health [136].

Essential fatty acids, particularly Omega-3s, are essential for maintaining a balanced inflammatory response. They exert anti-inflammatory effects by producing specialized mediators such as eicosanoids and resolvins, which help resolve inflammation and promote healing. Omega-3 fatty acids, including EPA and DHA, are converted into anti-inflammatory eicosanoids through the action of enzymes like cyclooxygenase (COX) and lipoxygenase (LOX). These mediators counteract the pro-inflammatory eicosanoids derived from arachidonic acid, an Omega-6 fatty acid, which can trigger chronic inflammation when consumed excessively. A deficiency in EFAs disrupts this delicate balance, increasing pro-inflammatory cytokines and mediators, contributing to chronic inflammatory conditions such as arthritis and asthma. This shift toward a pro-inflammatory state is often marked by elevated levels of interleukin-6 (IL-6) and tumor necrosis factor-alpha (TNF-α), which further drive inflammation and tissue damage [31,123].

Moreover, EFAs play an essential role in immune function and regulation. Sufficient levels of Omega-3 fatty acids are essential for the proper functioning of immune cells, including macrophages, T cells, and B cells. They affect the genes’ expression in immune responses, including those producing cytokines and chemokines.

A deficiency in EFAs can impair the ability of immune cells to communicate effectively, leading to a weakened immune response. This impairment can increase susceptibility to infections and hinder recovery after illness. The enzyme Δ5-desaturase, responsible for converting ALA (alpha-linolenic acid) to EPA and DHA, is crucial in this context; inadequate Omega-3 levels due to poor dietary intake can lead to dysfunction in immune signaling pathways [137].

In conclusion, essential fatty acids regulate inflammation and modulate immune function. Their deficiency can lead to a pro-inflammatory state characterized by elevated cytokine levels and increased susceptibility to infections. It underscores the importance of adequate EFA intake to promote a healthy immune response and mitigate chronic inflammation.

A deficiency in Omega-3 fatty acids, particularly eicosapentaenoic acid and docosahexaenoic acid, can significantly contribute to dyslipidemia, characterized by an unfavorable lipid profile. This condition is marked by elevated triglyceride levels and reduced high-density lipoprotein (HDL) cholesterol, often called “good” cholesterol. The mechanisms underlying this dyslipidemia involve the disruption of lipid metabolism, where Omega-3 fatty acids play a critical role in modulating hepatic lipid synthesis and clearance. Omega-3s enhance the activity of lipoprotein lipase (LPL), an enzyme that facilitates the breakdown of triglycerides in the bloodstream. A deficiency in these fatty acids can decrease LPL activity, resulting in higher circulating triglyceride levels.

Additionally, Omega-3s promote gene expression in synthesizing HDL cholesterol, such as those coding for apolipoprotein A1 (ApoA1). Lower HDL cholesterol levels can impair the reverse cholesterol transport, increasing the risk of cardiovascular diseases like atherosclerosis. Omega-3 fatty acids protect against heart disease through various mechanisms, including reducing inflammation, improving endothelial function, and regulating heart rhythms. EPA and DHA exert their anti-inflammatory effects by inhibiting the production of pro-inflammatory cytokines such as interleukin-1 (IL-1) and tumor necrosis factor-alpha (TNF-α), which contribute to vascular inflammation and atherosclerosis. These fatty acids also enhance the production of nitric oxide (NO), a key endothelial function regulator, promoting vasodilation and improving blood flow. In terms of cardiac rhythm, Omega-3s help stabilize cell membranes and modulate ion channel activity, which reduces the risk of arrhythmias. It is particularly important for preventing sudden cardiac death, often caused by dangerous heart rhythm disturbances [138,139].

As a result, a deficiency in Omega-3 fatty acids can lead to dyslipidemia, characterized by elevated triglycerides and decreased HDL cholesterol levels, which further increases the risk of cardiovascular diseases. Omega-3s’ protective mechanisms include their roles in lipid metabolism, inflammation regulation, endothelial function, and heart rhythm stabilization. Ensuring adequate intake of these fatty acids is crucial for maintaining cardiovascular health and preventing heart-related conditions.

Essential fatty acids are essential for reproductive health and play significant roles in fertility for both men and women. A deficiency in these vital nutrients can adversely affect sperm quality and ovulation. In men, Omega-3 fatty acids, particularly eicosapentaenoic acid and docosahexaenoic acid, contribute to the structural integrity of sperm membranes, enhancing their fluidity and functionality. It is mediated by the enzyme phospholipase A2, which releases fatty acids from membrane phospholipids, influencing sperm motility and morphology. In women, Omega-3 fatty acids play an essential role in regulating ovulatory processes by helping to modulate the production of hormones involved in the menstrual cycle, such as estrogen and progesterone. A deficiency in these fatty acids can lead to hormonal imbalances, disrupting ovulation and increasing the risk of infertility [140,141].

Omega-3 fatty acids are important during pregnancy for fetal development, especially in the growth of the brain and eyes. The fetal brain contains a high concentration of DHA, which is vital for neurogenesis and the formation of neuronal connections. A lack of Omega-3 intake during pregnancy can interfere with these processes, potentially leading to cognitive and developmental deficits in the child. Additionally, Omega-3 fatty acids help produce anti-inflammatory eicosanoids, which are important for supporting a healthy pregnancy. These eicosanoids regulate the immune response and prevent excessive inflammation, reducing the risks of complications such as preterm birth and preeclampsia. Omega-3s also affect gene expression in placental function and fetal growth, influencing birth weight and overall health outcomes. A deficiency in Omega-3 fatty acids can lead to fertility issues and complications during pregnancy, highlighting the need for adequate intake of these essential fatty acids for reproductive health and proper fetal development [142,143].

Moreover, Omega-3 fatty acids are essential for maintaining insulin sensitivity and regulating glucose metabolism. They improve insulin action in target tissues like muscle and adipose tissue by enhancing signaling pathways associated with insulin receptors. Omega-3s increase the expression of glucose transporter type 4 (GLUT4) through mechanisms involving the peroxisome proliferator-activated receptor gamma (PPAR-γ), a key transcription factor that enhances insulin sensitivity. When Omega-3 levels are low, especially in a diet high in Omega-6 fatty acids, the balance of fatty acid composition in cell membranes is disrupted. This imbalance can lead to an overproduction of pro-inflammatory mediators derived from Omega-6 fatty acids, contributing to systemic inflammation and insulin resistance. Chronic inflammation impairs insulin signaling, reducing glucose uptake and increasing the risk of developing type 2 diabetes [144].

Additionally, Omega-3 deficiency is linked to metabolic dysfunction, which can lead to the accumulation of visceral fat and a higher risk of metabolic syndrome, characterized by obesity, dyslipidemia, and hypertension. Omega-3s activate lipoprotein lipase (LPL), a key enzyme that facilitates the uptake of fatty acids into adipocytes while also promoting fat breakdown by enhancing hormone-sensitive lipase (HSL) activity [145].

PUFAs play a significant role in modulating gut microbiota, which is increasingly recognized as a key factor in the pathogenesis of metabolic syndrome. Emerging evidence suggests that PUFAs influence gut microbiota composition by promoting a eubiotic state characterized by the proliferation of beneficial bacterial strains, such as *Lactobacillus* and *Bifidobacterium* while inhibiting the growth of pathogenic microorganisms associated with dysbiosis. This shift toward eubiosis can help regulate gut barrier integrity, reduce intestinal permeability, and diminish systemic low-grade inflammation—a hallmark of metabolic diseases like type-2 diabetes mellitus, hyperlipidemia, and non-alcoholic fatty liver disease. On the other hand, Western dietary patterns, typically low in PUFAs and high in saturated fats and refined carbohydrates, exacerbate gut dysbiosis, creating an environment that fosters chronic inflammation and insulin resistance. By restoring microbial balance, PUFA-rich foods may mitigate the harmful effects of dysbiosis and attenuate the progression of metabolic syndrome. Moreover, these findings generate avenues for nutritional interventions to integrate PUFA-rich foods (such as fatty fish, flaxseeds, and walnuts) into dietary strategies supporting gut health and reducing the burden of metabolic disorders. This emerging understanding underscores the dual benefits of PUFAs: direct metabolic effects and their influence on gut microbiota composition, providing new opportunities for precision nutrition in managing metabolic syndrome [146,147].

Biochemically, PUFAs influence gut microbial dynamics through their structural and functional properties as signaling molecules, precursors for bioactive compounds, and modulators of membrane fluidity. Beneficial bacterial genera such as *Lactobacillus* and *Bifidobacterium* contribute to producing short-chain fatty acids (SCFAs) like butyrate, acetate, and propionate through the fermentation of dietary fibers. SCFAs, in turn, exert anti-inflammatory effects by activating G-protein coupled receptors (GPCRs) on intestinal epithelial cells and immune cells, suppressing nuclear factor kappa B (NF-κB) signaling pathways. This reduces the production of pro-inflammatory cytokines such as interleukin-6 (IL-6) and tumor necrosis factor-alpha (TNF-α), thereby mitigating chronic inflammation, a central feature of metabolic syndrome [148].

In addition to promoting beneficial bacteria, PUFAs can suppress the growth of pathogenic bacteria linked to dysbiosis, such as those from the *Enterobacteriaceae* family, by disrupting bacterial cell membranes. The amphipathic nature of PUFAs allows them to integrate into bacterial phospholipid bilayers, increasing membrane fluidity and permeability and ultimately impairing bacterial survival. Furthermore, PUFAs can modulate the gut immune response by influencing the production of antimicrobial peptides, such as defensins, which further help maintain microbial balance.

The anti-inflammatory effects of PUFAs also extend beyond the gut. For instance, through enzymatic oxidation pathways, Omega-3 PUFAs are converted into specialized pro-resolving mediators (SPMs), including resolvins, protectins, and maresins. These SPMs actively resolve inflammation by limiting neutrophil infiltration, enhancing macrophage-mediated clearance of cellular debris, and promoting tissue repair. This is particularly relevant in metabolic syndrome, where chronic, low-grade systemic inflammation is a key driver of insulin resistance, hyperlipidemia, and non-alcoholic fatty liver disease [149].

Moreover, PUFAs play a role in preserving intestinal barrier integrity. They regulate the expression of tight junction proteins such as occludin and claudins, preventing increased intestinal permeability (“leaky gut”) often observed in dysbiosis. By reducing lipopolysaccharides (LPS) translocation from gram-negative bacteria into the systemic circulation, PUFAs lower endotoxemia and the subsequent activation of toll-like receptor 4 (TLR4)-mediated inflammatory pathways implicated in metabolic disturbances.

PUFA-rich diets also influence bile acid metabolism, further shaping gut microbial composition. Secondary bile acids, modulated by microbial enzymatic activity, act as signaling molecules through nuclear receptors like the farnesoid X receptor (FXR) and Takeda G-protein receptor 5 (TGR5). These pathways are essential for regulating glucose and lipid metabolism, often dysregulated in metabolic syndrome [150,151].

## 8. Conclusions

Individuals can better manage the balance of these essential fatty acids through careful dietary choices that emphasize Omega-3-rich foods and minimize excessive Omega-6 intake. This approach supports overall health and is important in reducing the risk of inflammation-related chronic diseases.

The Omega-3 acids are polyunsaturated fatty acids found in large amounts in marine lipids, chia seeds, flax, walnuts, canola, and soybeans. Diets that provide a good amount of these Omega-3-rich foods are the Mediterranean diet, the Paleolithic diet, and the Okinawan diet. Supplementing the diet with this acid type brings special benefits to the body and longevity. To prevent metabolic diseases, an important role is also played by reducing the intake of saturated fats by replacing them with unsaturated ones corresponding to the body’s energy needs.

In conclusion, PUFAs are essential in maintaining and enhancing human health, offering wide-ranging benefits that extend from cardiovascular protection to anti-inflammatory and neuroprotective effects. As vital components of cell membranes and precursors to bioactive molecules, PUFAs are integral to numerous physiological processes. Their influence on reducing the risk of chronic diseases underscores the importance of adequate Omega-3 and Omega-6 fatty acids in modern diets. However, balancing these fatty acids is vital, as an imbalance may contribute to adverse health outcomes. Given the shift towards highly processed diets in contemporary society, PUFA-rich foods, such as fatty fish, nuts, seeds, and plant oils, is essential for optimizing health and preventing disease. As dietary practices evolve, focusing on the quality and composition of fats consumed will be vital in promoting long-term well-being. The profound intersection of PUFAs and polyphenols is a testament to the elegance of natural design, where nutritional complexity meets functional purpose. Harnessing this synergy in dietary frameworks requires robust research and a reimagining of food culture—one that values the interconnectedness of health, sustainability, and tradition. In the pursuit of this integrative approach, we not only enrich our lives but also contribute to the preservation of the planet for future generations. Thus, every meal becomes a step toward a healthier self and a healthier Earth, merging nourishment with stewardship in a meaningful and transformative way.

## Figures and Tables

**Table 1:** Polyunsaturated fatty acids with biological importance.

Acid Name	Short Formula	Chemical Structure	Sources and Benefits
Linoleic Acid (LA)	18:2 (Δ ^9Z,12Z^ /n-6)	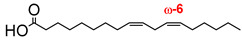	Linoleic acid (LA) is commonly found in plant-based oils, such as sunflower, safflower, soybean, corn, and canola. Additionally, it is present in foods like walnuts, flaxseeds, and sunflower seeds. LA, an essential Omega-6 fatty acid, supports various physiological functions. It supports the integrity of cell membranes, contributes to skin health, and acts as a foundational element for synthesizing other important bioactive molecules [8].
α-Linolenic Acid (ALA)	18:3 (Δ^9Z,12Z,15Z^ /n-3)	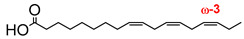	ALA is found primarily in plant-based foods: leafy green vegetables, flaxseeds and flaxseed oil, walnuts, hemp seeds, chia seeds, canola oil, and soybean oil. ALA is fundamental to maintaining overall health, notably by reducing inflammation and promoting cardiovascular well-being [9].
Rumelenic Acid	18:3 (Δ^9Z,11E,13E^ /n-3)	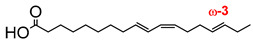	Rumelenic acid is found in the fat of ruminant animals like cows, sheep, and goats. Milk, cheese, and other dairy products from ruminants contain rumelenic acid. The fat content in beef and lamb contains rumelenic acid [10,11]. Though not as extensively researched as other fatty acids, it is part of the broader family of conjugated fatty acids, which have potential health benefits, particularly in areas like inflammation and metabolic health.
α-Eleostearic Acid	18:3 (Δ^9E,11E,13E^ /n-5)	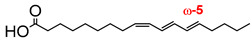	Bitter gourd oil contains 30–50% α-eleostearic acid. α-Eleostearic acid is primarily found in certain plant oils, notably in tung oil (from the tung tree seeds, *Vernicia fordii*). It is also present in some other plant oils but in smaller quantities. It has potential health benefits, including anti-inflammatory and antioxidant effects. Further research is needed to fully understand its roles and benefits in human health [12,13].
β-Eleostearic Acid	18:3 (Δ^9E,11Z,13E^ /n-5)	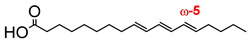	Present in tung oil (from the seeds of the tung tree, *Vernicia fordii*), which is rich in both α- and β-eleostearic acids. Tung oil is the primary natural source of β-eleostearic acid. It offers several potential health benefits, including anti-cancer, anti-inflammatory, and antioxidant effects. It may inhibit tumor growth, reduce inflammation, and neutralize free radicals, contributing to a lower risk of chronic diseases. Additionally, β-eleostearic acid shows promise in improving metabolic health by enhancing lipid profiles and regulating blood glucose levels, which could aid in managing cardiovascular diseases and diabetes. Emerging research also suggests its potential to support skin health, promote wound healing, and offer neuroprotective and anti-obesity effects [14].
Catalpic Acid	18:3 (Δ^9E,11E,13E^ /n-5)	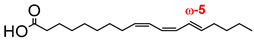	Catalpic acid is found in the seeds of certain plants, particularly in catalpa seeds from the Catalpa tree (*Catalpa* spp.). Its unique arrangement of double bonds gives it potential anti-inflammatory and antioxidant properties. Catalpic acid may also support cardiovascular health by improving lipid profiles, potentially lowering LDL cholesterol and triglycerides while increasing HDL cholesterol. Additionally, research suggests that catalpic acid could have anti-cancer effects by inhibiting the growth of certain cancer cells and inducing apoptosis [15].
Punicic Acid	18:3 (Δ^9Z,11E,13Z^ /n-5)	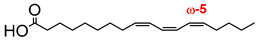	Punicic acid is predominantly found in the oil extracted from pomegranate seeds (*Punica granatum*) [16]. It has potential benefits for cardiovascular health and metabolic conditions.
γ-Linolenic Acid (GLA)	18:3 (Δ^9Z,11Z,13Z^ /n-6)	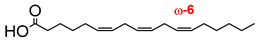	Gamma-linolenic acid (GLA) is in varying concentrations across several natural oils. Evening primrose oil (*Oenothera biennis* L.) offers 8–12% GLA, while hempseed oil (*Cannabis sativa* L.) contains approximately 6–8%. Among the richest sources, borage oil is notable for its high GLA content, ranging from 17–25%, followed by blackcurrant seed oil, which provides roughly 15–20% [17]. GLA is a beneficial Omega-6 fatty acid with anti-inflammatory properties, mainly from plant-based oils. It can support skin health, hormonal balance, and inflammatory conditions, making it a valuable supplement for many people [18].
α-Calendic Acid	18:3 (Δ^8Z,10E,12Z^ /n-6)	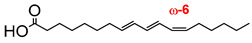	α-Calendic Acid is found primarily in the seed oils of certain plants: marigold (*Calendula officinalis*) seeds (marigold seed oil contains more than 30% of the total fatty acids), flaxseed oil, or some other plants in the *Asteraceae* family in smaller amounts. It has potential anti-inflammatory, antioxidant, and possibly anti-cancer properties, although more research is required to understand its benefits [19] fully.
β-Calendic Acid	18:3 (Δ^8E,10E,12Z^ /n-6)	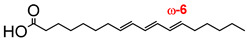	Similar to α-calendic acid, β-calendic acid is primarily found in the seeds of certain plants, particularly those from the *Asteraceae* family. The calendula plant is a well-known source of α- and β-calendic acids. Calendula seed oil contains more than 55% of the total fatty acids. While research on β-calendic acid is limited, it is believed to have anti-inflammatory, antioxidant, and potentially anti-cancer properties. However, more studies are needed to understand its effects and potential applications [19] fully.
Jacaric Acid	18:3 (Δ^9Z,11E,13Z^ /n-6)	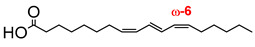	Jacaranda seed oil contains more 35% of the total fatty acids. The research on jacaric acid is still emerging, and much of the focus has been on its potential as an anti-cancer agent. Further studies are needed to fully understand its mechanism of action, therapeutic potential, and safety in humans [20].
Pinolenic Acid	18:3 (Δ^5Z,9Z,12Z^ /n-6)	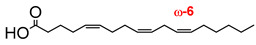	Pinolenic acid is derived mainly from the seeds of pine trees: Korean pine nuts (*Pinus koraiensis*), Siberian pine nuts (*Pinus sibirica*), and other pine species also contain pinolenic acid in their seeds [8]. Pinolenic acid has been studied for its potential health benefits, including appetite suppression, improved cardiovascular health, anti-inflammatory effects, and antioxidant activity. While research is still being conducted, pinolenic acid is promising as a natural supplement for weight management and heart health [21].
Stearidonic Acid (SDA)	18:4 (Δ^6Z,9Z,12Z,15Z^ /n-3)	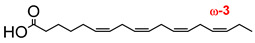	SDA is found in various plant and seed oils and marine sources: echium oil (*Echium plantagineum*), blackcurrant seed oil, hemp seed oil, borage oil, and evening primrose oil [22]. Research on SDA continues to expand, particularly concerning its role as a sustainable and plant-based source of Omega-3 fatty acids. It is being studied for its effects on heart health, inflammation, and overall well-being. SDA-rich oils are also becoming more common in supplements and functional foods to increase Omega-3 intake without relying on fish oil. SDA is valued for its ability to be more effectively converted into EPA in the body than ALA [23].
α-Parinaric Acid	18:4 (Δ^9Z,11E,13E,15Z^ /n-3)	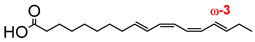	α-Parinaric acid is primarily found in the Parinari plant (*Parinari laurina*) seeds, which are native to tropical regions. While α-Parinaric acid is not a major dietary fatty acid, its unique chemical properties make it valuable for research in membrane biology, oxidative stress, and lipid biophysics. Its fluorescence properties are especially useful in understanding lipids’ behavior in different environmental conditions [24].
β-Parinaric Acid	18:4 (Δ^9Z,11E,13E,15E^ /n-3)	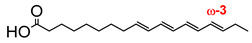	Like α-Parinaric acid, β-Parinaric acid is found in the seeds of the *Parinari* plant species. Although not a major component of the human diet, its role in scientific studies makes it a valuable tool for exploring the complexities of lipid biochemistry and cellular function [24].
Eicosadienoic Acid	20:2 (Δ^7Z,10Z^ /n-6)	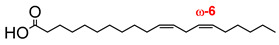	Eicosadienoic acid can be found in trace amounts in several plant oils: corn and soybean. It is present in small quantities in some animal tissues [25]. Eicosadienoic acid plays a role in lipid metabolism and cell membrane function and may influence inflammatory and cardiovascular processes. Further research is needed to fully understand its health implications and the balance of Omega-6 fatty acids in the diet [26].
Eicosatrienoic Acid (ETA)	20:3 (Δ^5Z,8Z,11Z^ /n-3)	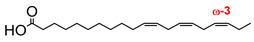	Eicosatrienoic acid (ETA) is found in small amounts in certain plant oils: evening primrose and borage oil. It can be synthesized as an intermediate during the conversion of alpha-linolenic acid (ALA) into longer-chain Omega-3s like EPA and DHA. ETA is involved in eicosanoid synthesis, influencing inflammation and cell membrane function. While research specifically targeting ETA is limited, its role in metabolic pathways suggests it may affect health and nutrition. Further studies are needed to elucidate its benefits and applications [27] entirely.
Dihomo-γ-linolenic Acid (DGLA)	20:3 (Δ^8Z,11Z,14Z^ /n-6)	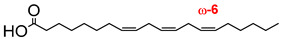	LA is a metabolic precursor of dihomo-γ-linolenic acid (DGLA). DGLA is found in trace amounts in oils like borage oil, evening primrose oil, and black currant seed oil. It can also be found in certain animal tissues, although it is less prominent than other fatty acids. DGLA has significant roles in modulating inflammation, supporting cardiovascular health, and improving skin conditions. Ongoing research continues to explore its full range of health benefits and applications [28].
Sciadonic Acid	20:3 (Δ^5Z,8Z,11Z,14Z^ /n-6)	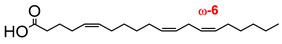	Sciadonic acid is primarily found in Catalpa tree seeds (*Catalpa* spp.). It plays a role in lipid metabolism, cell membrane function, and the synthesis of bioactive lipids. Although less studied compared to other fatty acids, sciadonic acid may have implications for inflammation and cardiovascular health. Further research is needed to fully understand its health benefits and potential uses in nutrition and supplementation [29].
Eicosatrienoic Acid (ETE) or Mead Acid (MA)	20:3 (Δ^5Z,8Z,11Z^ /n-3)	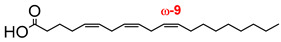	Because Mead acid is produced primarily under essential fatty acid deficiency conditions, it is generally not abundant in most diets. Adult mammals can synthesize MA from oleic acid. MA is typically found in small quantities in animal tissues, especially in species with low diets in EFAs. ETE plays a role in cell membrane function and can indicate essential fatty acid deficiency. It influences the production of eicosanoids involved in inflammation and immune responses. While its specific health impacts are less studied than other fatty acids, it is important to understand fatty acid metabolism and health conditions related to essential fatty acid imbalances [30].
Eicosatetraenoic Acid (ETA)	20:4 (Δ^5Z,8Z,11Z,14Z^ /n-3)	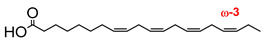	Eicosatetraenoic acid is typically produced within the body through the metabolic pathway of Omega-3 fatty acids. ETA contributes to lipid metabolism, supports cell membrane integrity, and participates in the production of eicosanoids, which play a critical role in regulating inflammation and immune responses. However, further research is required to fully understand its biological functions and explore its potential health benefits [31].
Arahidonic Acid (AA)	20:4 (Δ^5Z,8Z,11Z,14Z^ /n-6)	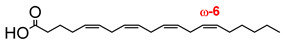	Arachidonic acid is obtained from poultry, animal organs, meat, fish, seafood, and eggs. It can be synthesized in the body from linoleic acid through elongation and desaturation processes. AA plays a central role in producing eicosanoids, influencing inflammation, immune responses, and cardiovascular health. It is also essential for brain function [32].
Eicosapentaenoic Acid (timnodonic acid, EPA)	20:5 (Δ^5Z,8Z,11Z,14Z,17Z^ /n-3)	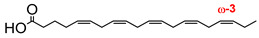	Eicosapentaenoic acid (EPA), sourced mainly from fish, is a significant Omega-3 fatty acid recognized for its health-promoting properties. It helps generate anti-inflammatory eicosanoids, supports heart health, decreases inflammation, and positively influences cognitive and mental function [33].
Bosseopentaenoic Acid	20:5 (Δ^5Z,8Z,11Z,14Z,17Z^ /n-6)	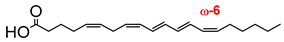	Bosseopentaenoic acid, sourced from marine organisms, contributes to producing anti-inflammatory eicosanoids. It is associated with potential advantages in promoting cardiovascular health and managing inflammation. However, specific research on bosseopentaenoic acid is limited, and most knowledge about its health implications is inferred from studies on related fatty acids [34].
Acid heneicosapentaenoic (HPA)	21:5 (Δ^5Z,8Z,11Z,14Z,17Z^ /n-3)	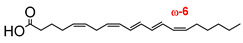	HPA is found in trace amounts in certain marine sources, particularly in some fish and algae. HPA plays a role in lipid metabolism and the production of eicosanoids, which are important for inflammation and immune responses. While specific research on HPA is limited, its potential benefits for inflammation and cardiovascular health are inferred from studies on similar fatty acids. Further research is needed to fully elucidate its health effects and applications [35].
Docosadienoic Acid	22:2 (Δ^7Z,10Z^ /n-6)	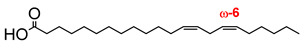	Docosadienoic acid is found in trace amounts in certain marine oils. It plays a role in lipid metabolism, cell membrane function, and eicosanoid synthesis. Although research on docosadienoic acid is limited, its involvement in these processes suggests potential health benefits related to inflammation and cardiovascular health. Further research is needed to fully understand its biological roles and health implications [36].
Adrenic Acid (AdA)	22:4 (Δ^5Z,8Z,11Z,14Z^ /n-6)	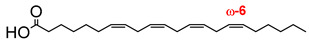	Adrenic acid is found in various foods, particularly animal products (meat, eggs, fish). AdA can be synthesized in the body from linoleic acid through elongation and desaturation processes. Adrenic acid plays a role in the synthesis of eicosanoids, influencing inflammation and immune responses. It is also essential for cell membrane function and may influence cardiovascular and neurological health. While research on adrenic acid is less extensive than other fatty acids, it is a significant component in understanding lipid metabolism and health [37].
Docosapentaenoic Acid (clupanodonic acid, DPA)	22:5 (Δ^7Z,10Z,13Z,16Z,19Z^ /n-3)	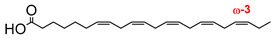	DPA is primarily found in marine organisms: fish and algae. DPA plays a role in the synthesis of eicosanoids, influencing inflammation and cardiovascular health. It is also essential for cell membrane function and may support neurological health [38,39].
Docosapentaenoic Acid (DPA)	22:5 (Δ^7Z,10Z,13Z,16Z,19Z^ /n-6)	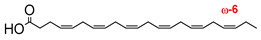	DPA (22:5 n-6) can be found in trace amounts in various foods, such as tissues of animals, certain fish, and seafood [38]. It can be synthesized in the body from other Omega-6 fatty acids, such as AA. DPA produces eicosanoids, affects inflammation and immune responses, and contributes to cell membrane function. Further research is needed to elucidate its health benefits and applications fully.
Docosahexaenoic Acid (cervonic acid, DHA)	22:6 (Δ^4Z,7Z,10Z,13Z,16Z,19Z^ /n-3)	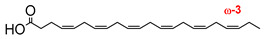	Docosahexaenoic acid (DHA), primarily derived from marine organisms, is also naturally present in breast milk. It plays a vital role in infant development, particularly in supporting brain and visual function [39]. Thus, DHA is essential for numerous physiological functions, including supporting cognitive abilities, maintaining visual health, and preserving the structural integrity of cell membranes. It also contributes to cardiovascular well-being and possesses anti-inflammatory effects. Furthermore, DHA is significant during pregnancy and breastfeeding, as it supports maternal health and fetal development. Extensive research supports DHA’s benefits across various aspects of health, making it an essential nutrient for overall well-being.
Tetracosatetraenoic Acid	24:4 (Δ^5Z,8Z,11Z,14Z^ /n-6)	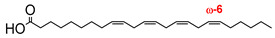	Tetracosatetraenoic acid is found in trace amounts in animal fats and marine sources and may also be present in certain plants. While less commonly studied than other fatty acids, its potential roles in lipid metabolism, cell membrane function, and eicosanoid synthesis suggest it could have physiological significance. Further research is needed to understand its health implications and contributions to overall well-being better [40].
Tetracosapentaenoic Acid (TPA) (ω-3)	24:5 (Δ^5Z,8Z,11Z,14Z,17Z^ /n-3)	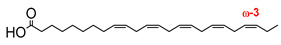	Tetracosapentaenoic acid (24:5 n-3, TPAn-3) and tetracosahexaenoic acid (24:6 n-3, THA) are believed to be important intermediates to docosahexaenoic acid (DHA, 22:6 n-3) synthesis [41]. TPA is present in trace amounts in certain marine sources and algae. TPA plays a role in lipid metabolism, eicosanoid production, and cell membrane function. While specific research on TPA is less extensive than EPA and DHA, it is expected to share similar benefits related to cardiovascular health, inflammation, and neurological function. Further research is needed to elucidate its health benefits and applications fully.
Tetracosapentaenoic Acid (ω-6)	24:5 (Δ^5Z,8Z,11Z,14Z,17Z^ /n-6)	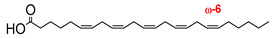	Tetracosapentaenoic acid (ω-6) is a relatively rare fatty acid in the human diet, occurring only in small quantities. It can be found in trace amounts within animal fats and plant-derived oils. TPA plays a role in lipid metabolism, eicosanoid production, and cell membrane function [42].
Tetracoxahexaenoic Acid (nisinic acid, THA)	24:6 (Δ^5Z,8Z,11Z,14Z,17Z,20Z^ /n-3)	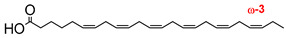	THA plays a role in lipid metabolism, eicosanoid synthesis, and cell membrane function. Further research is needed to understand its health benefits and applications fully. It is found in trace amounts in some marine sources and algae [43].

Legend: Δ (delta) notation: describes the positions of double bonds from the carboxyl (COOH) end; n (Omega) notation: identifies the position of the first double bond from the methyl (CH3) end of the fatty acid chain, classifying fatty acids such as Omega-3 and Omega-6; E notation: indicates that the double bond is in the trans configuration, where the hydrogen atoms are positioned on opposite sides of the double bond; Z notation: indicates a cis double bond, where the hydrogen atoms are positioned on the same side of the double bond; the numbers (4–20) specify the exact carbon atoms where the double bonds are located.

**Table 2:** The fat composition of various food sources.

Type of Food	Saturated Fatty Acids %	Monounsaturated Fatty Acids %	Polyunsaturated Fatty Acids %	Reference
Virgin Olive oil	14.27–18.30	67.58–76.37	7.91–14.39	[66]
Extra Virgin Olive Oil	14.81–18.01	66.30–74.30	10.90–15.90	[67]
Rapeseed oil	7.3	59.7	26.9	[68]
Virgin Hazelnut oil	8.3	75.4	13.3	[68]
Virgin Avocado oil	17.9	65.2	11.0	[68]
Virgin Argan oil	17.6	44.8	33.5	[68]
Virgin Grapeseed oil	11.2	19.5	63.6	[68]
Sunflower oil	10.9	30.8	54.6	[68]
Virgin Sesame oil	14.9	40.2	40.4	[68]
Virgin Coconut Oil	90.896	9.014	2.544	[69]
Cow Cheese	58.37–60.81	27.11–28.56	2.84–3.73	[70]
Sheep Cheese	54.60–60.65	21.05–28.52	3.92–4.65	[70]
Goat Cheese	52.72–61.64	21.41–30.56	2.99–4.24	[70]
Atlantic salmon	20.84 ± 1.14	46.56 ± 3.81	30.95 ± 3.35	[71]
Chicken egg	29.66 ± 3.26	39.07 ± 1.40	31.28 ± 3.83	[72]
Goose egg	34.17 ± 1.80	52.01 ± 1.53	13.84 ± 1.74	[72]
Duck egg	31.85 ± 0.35	52.49 ± 5.64	15.66 ± 5.57	[72]
Peanut	20.7 ± 5.7	60.6 ± 13.2	17.9 ± 8.6	[73]
Hazelnut	8.7 ± 1.1	79.6 ± 7.8	10.8 ± 8.7	[73]
Almond	8.9 ± 0.5	75.6 ± 2.5	14.7 ± 2.4	[73]
Pistachio	6.5 ± 1.8	30.1 ± 5.4	13.2 ± 3.5	[73]
Sesame	8.7 ± 1.2	22.7 ± 2.9	18.4 ± 2.7	[73]

**Table 3:** Summary of clinical studies on the health benefits of PUFAs.

Study Type	Nature of Participants	Duration Administered	Type and Dose of Fatty Acids Administered	Main Findings	References
Randomized, double-blind trial, controlled, crossover intervention trial	Hypercholesterolemic adults	12 weeks	ω-3 fish oil (1000 mg/day)	Improved triglycerides, LDL-cholesterol, and HDL-cholesterol concentration, as well as diastolic blood pressure (DBP)	[101]
Randomized, controlled trial	Impaired glucose metabolism participants	12 weeks	Fatty fish three times a week and bilberries in three portions per day (300 g)	Improved glucose metabolism and altered the lipidomic profile	[102]
Cross-sectional study, randomized, controlled trial	Healthy and glucose-intolerant subjects glucose and insulin homeostasis parameters in a large American sample	7 years	Dietary patterns (DP) heavily loaded with carbohydrate, SFA, PUFA, protein, total fat and MUFA	Favorable effects on insulin sensitivity and glucose tolerance	[103]
Double-blind controlled study, randomized	Subjects with highly elevated blood levels of triglycerides (≥500 mg/dL)	12 weeks	(i) 4 g/day supplementation of EPA ethyl ester; (ii) 2 g/day supplementation of EPA ethyl ester or; (iii) placebo	ω-3 PUFA supplementation can be helpful to counteract hypertriglyceridemia	[104]
Randomized, controlled, crossover, and prospective clinical study	Adults with hypertriglyceridemia were divided into groups: high-cardiovascular-risk (CV) patients and low-risk CV patient	12 weeks	ω-3 PUFA (1800 mg/day) or ciprofibrate (100 mg/day)	ω-3 PUFA improved arterial stiffness and endothelial function.	[105]
Randomized, double-blind, controlled trial	Chronic dialysis patients from Denmark	3 months	2 g marine ω-3 PUFA per day	ω-3 PUFA could contribute to vagal modulation that could be protective against malignant ventricular arrhythmias	[106]
Randomized double-blind study	Healthy young men and women to evaluate the effects of oral supplementation	12 weeks	3 g/day of (i) EPA, (ii) DHA, or (iii) olive oil	DHA supplementation may represent valid support for patients with high chronic BP	[107]
Randomized, double-blind, placebo-controlled trial	Patients with chronic heart failure	A median of 3.9 years	1 g/day of (i) ω-3 PUFA or (ii) placebo,	ω-3 PUFA supplementation may provide a small benefit in terms of mortality and hospitalization for CV reasons in heart failure patients	[108]
Randomized, controlled, double-blind study	Patients after acute MI (myocardial infarction)	6 months follow-up	(i) ω-3 PUFA supplementation (4 g/day) or (ii) placebo and underwent baseline assessment by CMR (Cardiac Magnetic Resonance imaging) 4–28 days after MI	PUFAs have an important effect on phenotypes of myocardial tissue after MI	[109]

## Data Availability

The original contributions presented in the study are included in the article; further inquiries can be directed to the corresponding authors.

## References

[1] Sokoła-Wysoczańska E., Wysoczański T., Wagner J., Czyż K., Bodkowski R., Lochyński S., Patkowska-Sokoła B. (2018). Polyunsaturated Fatty Acids and Their Potential Therapeutic Role in Cardiovascular System Disorders-A Review. Nutrients.

[2] Kapoor B., Kapoor D., Gautam S., Singh R., Bhardwaj S. (2021). Dietary Polyunsaturated Fatty Acids (PUFAs): Uses and Potential Health Benefits. Curr. Nutr. Rep..

[3] Shahidi F., Ambigaipalan P. (2018). Omega-3 Polyunsaturated Fatty Acids and Their Health Benefits. Annu. Rev. Food Sci. Technol..

[4] Bentsen H. (2017). Dietary polyunsaturated fatty acids, brain function and mental health. Microb. Ecol. Health Dis..

[5] Fillmore N., Mori J., Lopaschuk G.D. (2014). Mitochondrial fatty acid oxidation alterations in heart failure, ischaemic heart disease and diabetic cardiomyopathy. Br. J. Pharmacol..

[6] Li M., Liu Y., Li Q., Yang M., Pi Y., Yang N., Zheng Y., Yue X. (2020). Comparative exploration of free fatty acids in donkey colostrum and mature milk based on a metabolomics approach. J. Dairy Sci..

[7] Sioen I., van Lieshout L., Eilander A., Fleith M., Lohner S., Szommer A., Petisca C., Eussen S., Forsyth S., Calder P.C. (2017). Systematic Review on N-3 and N-6 Polyunsaturated Fatty Acid Intake in European Countries in Light of the Current Recommendations—Focus on Specific Population Groups. Ann. Nutr. Metab..

[8] Burns-Whitmore B., Froyen E., Heskey C., Parker T., San Pablo G. (2019). Alpha-Linolenic and Linoleic Fatty Acids in the Vegan Diet: Do They Require Dietary Reference Intake/Adequate Intake Special Consideration?. Nutrients.

[9] Saini R.K., Prasad P., Sreedhar R.V., Akhilender Naidu K., Shang X., Keum Y.S. (2021). Omega-3 Polyunsaturated Fatty Acids (PUFAs): Emerging Plant and Microbial Sources, Oxidative Stability, Bioavailability, and Health Benefits-A Review. Antioxidants.

[10] Addis M., Cabiddu A., Pinna G., Decandia M., Piredda G., Pirisi A., Molle G. (2005). Milk and Cheese Fatty Acid Composition in Sheep Fed Mediterranean Forages with Reference to Conjugated Linoleic Acid cis-9,trans-11. J. Dairy Sci..

[11] Alves S.P., Bessa R.J.B. (2007). Identification ofcis-12,cis-15 octadecadienoic acid and other minor polyenoic fatty acids in ruminant fat. Eur. J. Lipid Sci. Technol..

[12] Kung W.M., Lin M.S. (2021). Beneficial Impacts of Alpha-Eleostearic Acid from Wild Bitter Melon and Curcumin on Promotion of CDGSH Iron-Sulfur Domain 2: Therapeutic Roles in CNS Injuries and Diseases. Int. J. Mol. Sci..

[13] Gómez-Cortés P., Tyburczy C., Brenna J.T., Juárez M., de la Fuente M.A. (2009). Characterization ofcis-9trans-11trans-15 C18:3 in milk fat by GC and covalent adduct chemical ionization tandem MS. J. Lipid Res..

[14] Cui P., Lin Q., Fang D., Zhang L., Li R., Cheng J., Gao F., Shockey J., Hu S., Lü S. (2018). Tung Tree (Vernicia fordii, Hemsl.) Genome and Transcriptome Sequencing Reveals Co-Ordinate Up-Regulation of Fatty Acid β-Oxidation and Triacylglycerol Biosynthesis Pathways During Eleostearic Acid Accumulation in Seeds. Plant Cell Physiol..

[15] Oh Y., Lee D., Park S., Kim S.H., Kang K.S. (2021). The Chemical Constituents from Fruits of Catalpa bignonioides Walt. and Their α-Glucosidase Inhibitory Activity and Insulin Secretion Effect. Molecules.

[16] Koba K., Yanagita T. (2011). Potential Health Benefits of Pomegranate (*Punica granatum*) Seed Oil Containing Conjugated Linolenic Acid. Nuts and Seeds in Health and Disease.

[17] Rezapour-Firouzi S. (2017). Herbal Oil Supplement With Hot-Nature Diet for Multiple Sclerosis. Nutrition and Lifestyle in Neurological Autoimmune Diseases.

[18] Timoszuk M., Bielawska K., Skrzydlewska E. (2018). Evening Primrose (*Oenothera biennis*) Biological Activity Dependent on Chemical Composition. Antioxidants.

[19] Dulf F.V., Pamfil D., Baciu A.D., Pintea A. (2013). Fatty acid composition of lipids in pot marigold (*Calendula officinalis* L.) seed genotypes. Chem. Cent. J..

[20] Van Nieuwenhove C.P., Del Huerto Moyano A., Van Nieuwenhove G.A., Molina V., Luna Pizarro P. (2022). Jacaranda oil administration improves serum biomarkers and bioavailability of bioactive conjugated fatty acids, and alters fatty acid profile of mice tissues. Lipids.

[21] Takala R., Ramji D.P., Choy E. (2023). The Beneficial Effects of Pine Nuts and Its Major Fatty Acid, Pinolenic Acid, on Inflammation and Metabolic Perturbations in Inflammatory Disorders. Int. J. Mol. Sci..

[22] Harris W.S. (2012). Stearidonic Acid–Enhanced Soybean Oil: A Plant-Based Source of (n-3) Fatty Acids for Foods. J. Nutrition.

[23] Balić A., Vlašić D., Žužul K., Marinović B., Bukvić Mokos Z. (2020). Omega-3 Versus Omega-6 Polyunsaturated Fatty Acids in the Prevention and Treatment of Inflammatory Skin Diseases. Int. J. Mol. Sci..

[24] Puri R., Choudhary A.K., Barman P., Mishra G., Geeta R. (2022). Two unusual conjugated fatty acids, parinaric acid and α-eleostearic acid, are present in several Impatiens species, but not in congener Hydrocera triflora. Physiol. Mol. Biol. Plants.

[25] Huang Y.-S., Huang W.-C., Li C.-W., Chuang L.-T. (2011). Eicosadienoic acid differentially modulates production of pro-inflammatory modulators in murine macrophages. Mol. Cell. Biochem..

[26] Rahim M.A., Ayub H., Sehrish A., Ambreen S., Khan F.A., Itrat N., Nazir A., Shoukat A., Shoukat A., Ejaz A. (2023). Essential Components from Plant Source Oils: A Review on Extraction, Detection, Identification, and Quantification. Molecules.

[27] Abedi E., Sahari M.A. (2014). Long-chain polyunsaturated fatty acid sources and evaluation of their nutritional and functional properties. Food Sci. Nutr..

[28] Knez M., Stangoulis J., Glibetic M., Tako E. (2017). The Linoleic Acid: Dihomo-γ-Linolenic Acid Ratio (LA:DGLA)—An Emerging Biomarker of Zn Status. Nutrients.

[29] Pédrono F., Boulier-Monthéan N., Boissel F., Ossemond J., Viel R., Fautrel A., Marchix J., Dupont D. (2020). Sciadonic acid derived from pine nuts as a food component to reduce plasma triglycerides by inhibiting the rat hepatic Δ9-desaturase. Sci. Rep..

[30] Kawashima H., Yoshizawa K. (2023). The physiological and pathological properties of Mead acid, an endogenous multifunctional n-9 polyunsaturated fatty acid. Lipids Health Dis..

[31] Calder P.C. (2010). Omega-3 fatty acids and inflammatory processes. Nutrients.

[32] Tallima H., El Ridi R. (2018). Arachidonic acid: Physiological roles and potential health benefits—A review. J. Adv. Res..

[33] Swanson D., Block R., Mousa S.A. (2012). Omega-3 fatty acids EPA and DHA: Health benefits throughout life. Adv. Nutr..

[34] Lu Y., Chen Y., Wu Y., Hao H., Liang W., Liu J., Huang R. (2019). Marine unsaturated fatty acids: Structures, bioactivities, biosynthesis and benefits. RSC Adv..

[35] Larsen L.N., Høvik K., Bremer J., Holm K.H., Myhren F., Børretzen B. (1997). Heneicosapentaenoate (21:5n−3): Its incorporation into lipids and its effects on arachidonic acid and eicosanoid synthesis. Lipids.

[36] Chen Y., Qiu X., Yang J. (2020). Comparing the In Vitro Antitumor, Antioxidant and Anti-Inflammatory Activities between Two New Very Long Chain Polyunsaturated Fatty Acids, Docosadienoic Acid (DDA) and Docosatrienoic Acid (DTA), and Docosahexaenoic Acid (DHA). Nutr. Cancer.

[37] Singh N., Barnych B., Wagner K.M., Wan D., Morisseau C., Hammock B.D. (2021). Adrenic Acid-Derived Epoxy Fatty Acids Are Naturally Occurring Lipids and Their Methyl Ester Prodrug Reduces Endoplasmic Reticulum Stress and Inflammatory Pain. ACS Omega.

[38] Zheng S., Chen T.C. (2016). Fish, Fish Oil, and Liver Cancer. Fish and Fish Oil in Health and Disease Prevention.

[39] Castillo F., Castillo-Ferrer F.-J., Cordobilla B., Domingo J.C. (2021). Inadequate Content of Docosahexaenoic Acid (DHA) of Donor Human Milk for Feeding Preterm Infants: A Comparison with Mother’s Own Milk at Different Stages of Lactation. Nutrients.

[40] Rizzo G., Baroni L., Lombardo M. (2023). Promising Sources of Plant-Derived Polyunsaturated Fatty Acids: A Narrative Review. Int. J. Environ. Res. Public Health.

[41] Metherel A.H., Domenichiello A.F., Kitson A.P., Lin Y.-H., Bazinet R.P. (2016). Serum n-3 Tetracosapentaenoic Acid and Tetracosahexaenoic Acid Increase Following Higher Dietary α-Linolenic Acid but not Docosahexaenoic Acid. Lipids.

[42] Ponnampalam E.N., Sinclair A.J., Holman B.W.B. (2021). The Sources, Synthesis and Biological Actions of Omega-3 and Omega-6 Fatty Acids in Red Meat: An Overview. Foods.

[43] Itoh T., Tomiyasu A., Yamamoto K. (2011). Efficient Synthesis of the Very-Long-Chain n-3 Fatty Acids, Tetracosahexaenoic Acid (C24:6n-3) and Tricosahexaenoic Acid (C23:6n-3). Lipids.

[44] Kaur N., Chugh V., Gupta A.K. (2014). Essential fatty acids as functional components of foods- a review. J. Food Sci. Technol..

[45] Simonetto M., Infante M., Sacco R.L., Rundek T., Della-Morte D. (2019). A Novel Anti-Inflammatory Role of Omega-3 PUFAs in Prevention and Treatment of Atherosclerosis and Vascular Cognitive Impairment and Dementia. Nutrients.

[46] Djuricic I., Calder P.C. (2021). Beneficial Outcomes of Omega-6 and Omega-3 Polyunsaturated Fatty Acids on Human Health: An Update for 2021. Nutrients.

[47] Tsoupras A., Brummell C., Kealy C., Vitkaitis K., Redfern S., Zabetakis I. (2022). Cardio-Protective Properties and Health Benefits of Fish Lipid Bioactives; The Effects of Thermal Processing. Mar. Drugs.

[48] Mititelu M., Nicolescu T.O., Ioniţă C.A., Nicolescu F. (2012). Study of Heavy Metals and Organic Polluants From Some Fisches of Danube River. J. Enviro. Prot. Ecol..

[49] Mititelu M., Moroșan E., Nicoară A.C., Secăreanu A.A., Musuc A.M., Atkinson I., Pandele Cusu J., Nițulescu G.M., Ozon E.A., Sarbu I. (2022). Development of Immediate Release Tablets Containing Calcium Lactate Synthetized from Black Sea Mussel Shells. Mar. Drugs.

[50] Mititelu M., Ghica M., Ionita A.C., Moroşan E. (2019). The influence of heavy metals contamination in soil on the composition of some wild edible mushrooms. Farmacia.

[51] Ioniță-Mîndrican C.-B., Mititelu M., Musuc A.M., Oprea E., Ziani K., Neacșu S.M., Grigore N.D., Negrei C., Dumitrescu D.-E., Mireșan H. (2022). Honey and Other Beekeeping Products Intake among the Romanian Population and Their Therapeutic Use. Appl. Sci..

[52] Pop A.L., Henteș P., Pali M.-A., Oșanu L., Ciobanu A., Nasui B.A., Mititelu M., Crișan S., Peneș O.N. (2022). Study regarding a new extended-release calcium ascorbate and hesperidin solid oral formulation. Farmacia.

[53] Mititelu M., Udeanu D.I., Docea A.O., Tsatsakis A., Calina D., Arsene A.L., Nedelescu M., Neacsu S.M., Velescu B.S., Ghica M. (2023). New method for risk assessment in environmental health: The paradigm of heavy metals in honey. Environ. Res..

[54] Napier J.A., Usher S., Haslam R.P., Ruiz-Lopez N., Sayanova O. (2015). Transgenic plants as a sustainable, terrestrial source of fish oils. Eur. J. Lipid Sci. Technol..

[55] Suárez-Medina M.D., Sáez-Casado M.I., Martínez-Moya T., Rincón-Cervera M.Á. (2024). The Effect of Low Temperature Storage on the Lipid Quality of Fish, Either Alone or Combined with Alternative Preservation Technologies. Foods.

[56] Perna M., Hewlings S. (2022). Saturated Fatty Acid Chain Length and Risk of Cardiovascular Disease: A Systematic Review. Nutrients.

[57] Ruiz M., Bodhicharla R., Svensk E., Devkota R., Busayavalasa K., Palmgren H., Ståhlman M., Boren J., Pilon M. (2018). Membrane fluidity is regulated by the C. elegans transmembrane protein FLD-1 and its human homologs TLCD1/2. eLife.

[58] Patterson E., Wall R., Fitzgerald G.F., Ross R.P., Stanton C. (2012). Health implications of high dietary omega-6 polyunsaturated Fatty acids. J. Nutr. Metab..

[59] D’Angelo S., Motti M.L., Meccariello R. (2020). ω-3 and ω-6 Polyunsaturated Fatty Acids, Obesity and Cancer. Nutrients.

[60] Smolińska K., Szopa A., Sobczyński J., Serefko A., Dobrowolski P. (2024). Nutritional Quality Implications: Exploring the Impact of a Fatty Acid-Rich Diet on Central Nervous System Development. Nutrients.

[61] Cena H., Calder P.C. (2020). Defining a Healthy Diet: Evidence for The Role of Contemporary Dietary Patterns in Health and Disease. Nutrients.

[62] DiNicolantonio J.J., O’Keefe J. (2021). The Importance of Maintaining a Low Omega-6/Omega-3 Ratio for Reducing the Risk of Autoimmune Diseases, Asthma, and Allergies. MO. Med..

[63] Yamashima T., Ota T., Mizukoshi E., Nakamura H., Yamamoto Y., Kikuchi M., Yamashita T., Kaneko S. (2020). Intake of ω-6 Polyunsaturated Fatty Acid-Rich Vegetable Oils and Risk of Lifestyle Diseases. Adv. Nutr..

[64] Kris-Etherton P.M., Krauss R.M. (2020). Public health guidelines should recommend reducing saturated fat consumption as much as possible: YES. Am. J. Clin. Nutr..

[65] Liu A.G., Ford N.A., Hu F.B., Zelman K.M., Mozaffarian D., Kris-Etherton P.M. (2017). A healthy approach to dietary fats: Understanding the science and taking action to reduce consumer confusion. Nutr. J..

[66] Revelou P.K., Xagoraris M., Alexandropoulou A., Kanakis C.D., Papadopoulos G.K., Pappas C.S., Tarantilis P.A. (2021). Chemometric Study of Fatty Acid Composition of Virgin Olive Oil from Four Widespread Greek Cultivars. Molecules.

[67] Di Serio M.G., Di Giacinto L., Di Loreto G., Giansante L., Pellegrino M., Vito R., Perri E. (2016). Chemical and sensory characteristics of Italian virgin olive oils from Grossa di Gerace cv. Eur. J. Lipid Sci. Technol..

[68] Vingering N., Oseredczuk M., du Chaffaut L., Ireland J., Ledoux M. (2010). Fatty acid composition of commercial vegetable oils from the French market analysed using a long highly polar column. OCL.

[69] Sabahannur S., Alimuddin S. (2022). Identification of Fatty Acids in Virgin Coconut Oil (VCO), Cocoa Beans, Crude Palm Oil (CPO), and Palm Kernel Beans Using Gas Chromatography. IOP Conf. Ser. Earth Environ. Sci..

[70] Paszczyk B., Łuczyńska J. (2020). The Comparison of Fatty Acid Composition and Lipid Quality Indices in Hard Cow, Sheep, and Goat Cheeses. Foods.

[71] Jensen I.J., Eilertsen K.E., Otnæs C.H.A., Mæhre H.K., Elvevoll E.O. (2020). An Update on the Content of Fatty Acids, Dioxins, PCBs and Heavy Metals in Farmed, Escaped and Wild Atlantic Salmon (*Salmo salar* L.) in Norway. Foods.

[72] Polat E.S., Citil O.B., Garip M. (2013). Fatty acid composition of yolk of nine poultry species kept in their natural environment. Anim. Sci. Pap. Rep..

[73] Vecka M., Staňková B., Kutová S., Tomášová P., Tvrzická E., Žák A. (2019). Comprehensive sterol and fatty acid analysis in nineteen nuts, seeds, and kernel. SN Appl. Sci..

[74] Clemente-Suárez V.J., Beltrán-Velasco A.I., Redondo-Flórez L., Martín-Rodríguez A., Tornero-Aguilera J.F. (2023). Global Impacts of Western Diet and Its Effects on Metabolism and Health: A Narrative Review. Nutrients.

[75] Lăcătușu C.M., Grigorescu E.D., Floria M., Onofriescu A., Mihai B.M. (2019). The Mediterranean Diet: From an Environment-Driven Food Culture to an Emerging Medical Prescription. Int. J. Environ. Res. Public Health.

[76] Frączek B., Pięta A., Burda A., Mazur-Kurach P., Tyrała F. (2021). Paleolithic Diet—Effect on the Health Status and Performance of Athletes?. Nutrients.

[77] Willcox D.C., Willcox B.J., Todoriki H., Suzuki M. (2009). The Okinawan diet: Health implications of a low-calorie, nutrient-dense, antioxidant-rich dietary pattern low in glycemic load. J. Am. Coll. Nutr..

[78] Meijaard E., Abrams J.F., Slavin J.L., Sheil D. (2022). Dietary Fats, Human Nutrition and the Environment: Balance and Sustainability. Front. Nutr..

[79] DiNicolantonio J.J., O’Keefe J.H. (2022). Monounsaturated Fat vs Saturated Fat: Effects on Cardio-Metabolic Health and Obesity. MO. Med..

[80] Alcorta A., Porta A., Tárrega A., Alvarez M.D., Vaquero M.P. (2021). Foods for Plant-Based Diets: Challenges and Innovations. Foods.

[81] Dhaka V., Gulia N., Ahlawat K.S., Khatkar B.S. (2011). Trans fats-sources, health risks and alternative approach—A review. J. Food Sci. Technol..

[82] Pipoyan D., Stepanyan S., Stepanyan S., Beglaryan M., Costantini L., Molinari R., Merendino N. (2021). The Effect of Trans Fatty Acids on Human Health: Regulation and Consumption Patterns. Foods.

[83] Anand S.S., Hawkes C., de Souza R.J., Mente A., Dehghan M., Nugent R., Zulyniak M.A., Weis T., Bernstein A.M., Krauss R.M. (2015). Food Consumption and its Impact on Cardiovascular Disease: Importance of Solutions Focused on the Globalized Food System: A Report From the Workshop Convened by the World Heart Federation. J. Am. Coll. Cardiol..

[84] Touvier M., da Costa Louzada M.L., Mozaffarian D., Baker P., Juul F., Srour B. (2023). Ultra-processed foods and cardiometabolic health: Public health policies to reduce consumption cannot wait. BMJ.

[85] Madureira Lima J., Rayner M., Breda J., Jewell J. (2022). The European Food Regulatory Environment Index: A tool to monitor progress in implementing food environment policies. Eur. J. Public Health.

[86] Hwalla N., Jaafar Z., Sawaya S. (2021). Dietary Management of Type 2 Diabetes in the MENA Region: A Review of the Evidence. Nutrients.

[87] Tarar O.M., Ahmed K.M., Nishtar N.A., Achakzai A.B.K., Gulzar Y., Delles C., Al-Jawaldeh A. (2020). Understanding the complexities of prevalence of trans fat and its control in food supply in Pakistan. J. Clin. Hypertens..

[88] Rashid A., Amjad S., Nishtar M.K., Nishtar N.A. (2020). Trans-Fatty Acid (TFA) elimination in Pakistan: A situational analysis. J. Pak. Med. Assoc..

[89] Popkin B.M., Reardon T. (2018). Obesity and the food system transformation in Latin America. Obes. Rev..

[90] Casari S., Di Paola M., Banci E., Diallo S., Scarallo L., Renzo S., Gori A., Renzi S., Paci M., de Mast Q. (2022). Changing Dietary Habits: The Impact of Urbanization and Rising Socio-Economic Status in Families from Burkina Faso in Sub-Saharan Africa. Nutrients.

[91] Boateng L., Ansong R., Owusu W.B., Steiner-Asiedu M. (2016). Coconut oil and palm oil’s role in nutrition, health and national development: A review. Ghana Med. J..

[92] Tu W.C., Cook-Johnson R.J., James M.J., Mühlhäusler B.S., Gibson R.A. (2010). Omega-3 long chain fatty acid synthesis is regulated more by substrate levels than gene expression. Prostaglandins Leukot. Essent. Fat. Acids.

[93] Takic M., Pokimica B., Petrovic-Oggiano G., Popovic T. (2022). Effects of Dietary α-Linolenic Acid Treatment and the Efficiency of Its Conversion to Eicosapentaenoic and Docosahexaenoic Acids in Obesity and Related Diseases. Molecules.

[94] Michaeloudes C., Christodoulides S., Christodoulou P., Kyriakou T.-C., Patrikios I., Stephanou A. (2023). Variability in the Clinical Effects of the Omega-3 Polyunsaturated Fatty Acids DHA and EPA in Cardiovascular Disease—Possible Causes and Future Considerations. Nutrients.

[95] Smith C.E., Follis J.L., Nettleton J.A., Foy M., Wu J.H., Ma Y., Tanaka T., Manichakul A.W., Wu H., Chu A.Y. (2015). Dietary fatty acids modulate associations between genetic variants and circulating fatty acids in plasma and erythrocyte membranes: Meta-analysis of nine studies in the CHARGE consortium. Mol. Nutr. Food Res..

[96] Petermann A.B., Reyna-Jeldes M., Ortega L., Coddou C., Yévenes G.E. (2022). Roles of the Unsaturated Fatty Acid Docosahexaenoic Acid in the Central Nervous System: Molecular and Cellular Insights. Int. J. Mol. Sci..

[97] Das U.N. (2021). Essential Fatty Acids and Their Metabolites in the Pathobiology of Inflammation and Its Resolution. Biomolecules.

[98] Sheppe A.E.F., Edelmann M.J. (2021). Roles of Eicosanoids in Regulating Inflammation and Neutrophil Migration as an Innate Host Response to Bacterial Infections. Infect. Immun..

[99] Sonnweber T., Pizzini A., Nairz M., Weiss G., Tancevski I. (2018). Arachidonic Acid Metabolites in Cardiovascular and Metabolic Diseases. Int. J. Mol. Sci..

[100] Glick N.R., Fischer M.H. (2013). The Role of Essential Fatty Acids in Human Health. J. Evid. Based Complement. Altern. Med..

[101] Shen T., Xing G., Zhu J., Zhang S., Cai Y., Li D., Xu G., Xing E., Rao J., Shi R. (2017). Effects of 12-week supplementation of marine Omega-3 PUFA-based formulation Omega3Q10 in older adults with prehypertension and/or elevated blood cholesterol. Lipids Health Dis..

[102] Lankinen M., Schwab U., Kolehmainen M., Paananen J., Poutanen K., Mykkänen H., Seppänen-Laakso T., Gylling H., Uusitupa M., Orešič M. (2011). Whole grain products, fish and bilberries alter glucose and lipid metabolism in a randomized, controlled trial: The Sysdimet study. PLoS ONE.

[103] Mazidi M., Kengne A.P., Mikhailidis D.P., Toth P.P., Ray K.K., Banach M. (2017). Dietary food patterns and glucose/insulin homeostasis: A cross-sectional study involving 24,182 adult Americans. Lipids Health Dis..

[104] Bays H.E., Ballantyne C.M., Kastelein J.J., Isaacsohn J.L., Braeckman R.A., Soni P.N. (2011). Eicosapentaenoic Acid Ethyl Ester (AMR101) Therapy in Patients With Very High Triglyceride Levels (from the Multi-center, plAcebo-controlled, Randomized, double-blINd, 12-week study with an open-label Extension [MARINE] Trial). Am. J. Cardiol..

[105] Casanova M.A., Medeiros F., Trindade M., Cohen C., Oigman W., Neves M.F. (2017). Omega-3 fatty acids supplementation improves endothelial function and arterial stiffness in hypertensive patients with hypertriglyceridemia and high cardiovascular risk. J. Am. Soc. Hypertens..

[106] Rantanen J., Riahi S., Johansen M., Schmidt E., Christensen J. (2018). Effects of Marine n-3 Polyunsaturated Fatty Acids on Heart Rate Variability and Heart Rate in Patients on Chronic Dialysis: A Randomized Controlled Trial. Nutrients.

[107] Lee J.B., Notay K., Klingel S., Chabowski A., Mutch D.M., Millar P.J. (2019). Docosahexaenoic acid reduces resting blood pressure but increases muscle sympathetic outflow compared to eicosapentaenoic acid in healthy men and women. Am. J. Physiol. Heart Circ. Physiol..

[108] Tavazzi L., Maggioni A.P., Marchioli R., Barlera S., Franzosi M.G., Latini R., Lucci D., Nicolosi G.L., Porcu M., Tognoni G. (2008). Effect of n-3 polyunsaturated fatty acids in patients with chronic heart failure (the GISSI-HF trial): A randomised, double-blind, placebo-controlled trial. Lancet.

[109] Heydari B., Abdullah S., Pottala J.V., Shah R., Abbasi S., Mandry D., Francis S.A., Lumish H., Ghoshhajra B.B., Hoffmann U. (2016). Effect of Omega-3 Acid Ethyl Esters on Left Ventricular Remodeling After Acute Myocardial Infarction: The OMEGA-REMODEL Randomized Clinical Trial. Circulation.

[110] Valentine R.C., Valentine D.L. (2004). Omega-3 fatty acids in cellular membranes: A unified concept. Prog. Lipid Res..

[111] de Carvalho C.C.C.R., Caramujo M.J. (2018). The Various Roles of Fatty Acids. Molecules.

[112] Dyall S.C. (2015). Long-chain omega-3 fatty acids and the brain: A review of the independent and shared effects of EPA, DPA and DHA. Front. Aging Neurosci..

[113] Dighriri I.M., Alsubaie A.M., Hakami F.M., Hamithi D.M., Alshekh M.M., Khobrani F.A., Dalak F.E., Hakami A.A., Alsueaadi E.H., Alsaawi L.S. (2022). Effects of Omega-3 Polyunsaturated Fatty Acids on Brain Functions: A Systematic Review. Cureus.

[114] Healy-Stoffel M., Levant B. (2018). N-3 (Omega-3) Fatty Acids: Effects on Brain Dopamine Systems and Potential Role in the Etiology and Treatment of Neuropsychiatric Disorders. CNS Neurol. Disord. Drug Targets.

[115] DiNicolantonio J.J., O’Keefe J.H. (2020). The Importance of Marine Omega-3s for Brain Development and the Prevention and Treatment of Behavior, Mood, and Other Brain Disorders. Nutrients.

[116] Liu Y.X., Yu J.H., Sun J.H., Ma W.Q., Wang J.J., Sun G.J. (2023). Effects of Omega-3 Fatty Acids Supplementation on Serum Lipid Profile and Blood Pressure in Patients with Metabolic Syndrome: A Systematic Review and Meta-Analysis of Randomized Controlled Trials. Foods.

[117] Howell G., Deng X., Yellaturu C., Park E.A., Wilcox H.G., Raghow R., Elam M.B. (2009). N-3 polyunsaturated fatty acids suppress insulin-induced SREBP-1c transcription via reduced trans-activating capacity of LXRalpha. Biochim. Biophys. Acta.

[118] Ipsen D.H., Lykkesfeldt J., Tveden-Nyborg P. (2018). Molecular mechanisms of hepatic lipid accumulation in non-alcoholic fatty liver disease. Cell Mol. Life Sci..

[119] Sherratt S.C.R., Libby P., Budoff M.J., Bhatt D.L., Mason R.P. (2023). Role of Omega-3 Fatty Acids in Cardiovascular Disease: The Debate Continues. Curr. Atheroscler. Rep..

[120] Stephenson J.A., Al-Taan O., Arshad A., Morgan B., Metcalfe M.S., Dennison A.R. (2013). The multifaceted effects of omega-3 polyunsaturated Fatty acids on the hallmarks of cancer. J. Lipids.

[121] D’Eliseo D., Velotti F. (2016). Omega-3 Fatty Acids and Cancer Cell Cytotoxicity: Implications for Multi-Targeted Cancer Therapy. J. Clin. Med..

[122] Botta M., Audano M., Sahebkar A., Sirtori C.R., Mitro N., Ruscica M. (2018). PPAR Agonists and Metabolic Syndrome: An Established Role?. Int. J. Mol. Sci..

[123] Calder P.C. (2013). Omega-3 polyunsaturated fatty acids and inflammatory processes: Nutrition or pharmacology?. Br. J. Clin. Pharmacol..

[124] Kalupahana N.S., Claycombe K.J., Moustaid-Moussa N. (2011). (n-3) Fatty acids alleviate adipose tissue inflammation and insulin resistance: Mechanistic insights. Adv. Nutr..

[125] Simard M., Julien P., Fradette J., Pouliot R. (2019). Modulation of the Lipid Profile of Reconstructed Skin Substitutes after Essential Fatty Acid Supplementation Affects Testosterone Permeability. Cells.

[126] Michalak M., Pierzak M., Kręcisz B., Suliga E. (2021). Bioactive Compounds for Skin Health: A Review. Nutrients.

[127] Gow R.V., Hibbeln J.R. (2014). Omega-3 fatty acid and nutrient deficits in adverse neurodevelopment and childhood behaviors. Child. Adolesc. Psychiatr. Clin. N. Am..

[128] Mariamenatu A.H., Abdu E.M. (2021). Overconsumption of Omega-6 Polyunsaturated Fatty Acids (PUFAs) versus Deficiency of Omega-3 PUFAs in Modern-Day Diets: The Disturbing Factor for Their “Balanced Antagonistic Metabolic Functions” in the Human Body. J. Lipids.

[129] Purnamawati S., Indrastuti N., Danarti R., Saefudin T. (2017). The Role of Moisturizers in Addressing Various Kinds of Dermatitis: A Review. Clin. Med. Res..

[130] Parke M.A., Perez-Sanchez A., Zamil D.H., Katta R. (2021). Diet and Skin Barrier: The Role of Dietary Interventions on Skin Barrier Function. Dermatol. Pract. Concept..

[131] Lin T.-K., Zhong L., Santiago J.L. (2018). Anti-Inflammatory and Skin Barrier Repair Effects of Topical Application of Some Plant Oils. Int. J. Mol. Sci..

[132] Huffman S.L., Harika R.K., Eilander A., Osendarp S.J. (2011). Essential fats: How do they affect growth and development of infants and young children in developing countries? A literature review. Matern. Child. Nutr..

[133] Uauy R., Dangour A.D. (2006). Nutrition in brain development and aging: Role of essential fatty acids. Nutr. Rev..

[134] Shindou H., Koso H., Sasaki J., Nakanishi H., Sagara H., Nakagawa K.M., Takahashi Y., Hishikawa D., Iizuka-Hishikawa Y., Tokumasu F. (2017). Docosahexaenoic acid preserves visual function by maintaining correct disc morphology in retinal photoreceptor cells. J. Biol. Chem..

[135] Welty F.K. (2023). Omega-3 fatty acids and cognitive function. Curr. Opin. Lipidol..

[136] Lauretani F., Bandinelli S., Bartali B., Cherubini A., Iorio A.D., Blè A., Giacomini V., Corsi A.M., Guralnik J.M., Ferrucci L. (2007). Omega-6 and omega-3 fatty acids predict accelerated decline of peripheral nerve function in older persons. Eur. J. Neurol..

[137] Gutiérrez S., Svahn S.L., Johansson M.E. (2019). Effects of Omega-3 Fatty Acids on Immune Cells. Int. J. Mol. Sci..

[138] Bayram S.Ş., Kızıltan G. (2024). The Role of Omega-3 Polyunsaturated Fatty Acids in Diabetes Mellitus Management: A Narrative Review. Curr. Nutr. Rep..

[139] Kobayashi Y., Fujikawa T., Haruna A., Kawano R., Ozawa M., Haze T., Komiya S., Suzuki S., Ohki Y., Fujiwara A. (2024). Omega-3 Fatty Acids Reduce Remnant-like Lipoprotein Cholesterol and Improve the Ankle–Brachial Index of Hemodialysis Patients with Dyslipidemia: A Pilot Study. Medicina.

[140] Salas-Huetos A., Arvizu M., Mínguez-Alarcón L., Mitsunami M., Ribas-Maynou J., Yeste M., Ford J.B., Souter I., Chavarro J.E. (2022). EARTH Study Team. Women’s and men’s intake of omega-3 fatty acids and their food sources and assisted reproductive technology outcomes. Am. J. Obstet. Gynecol..

[141] Ma X., Wu L., Wang Y., Han S., El-Dalatony M.M., Feng F., Tao Z., Yu L., Wang Y. (2022). Diet and human reproductive system: Insight of omics approaches. Food Sci. Nutr..

[142] Coletta J.M., Bell S.J., Roman A.S. (2010). Omega-3 Fatty acids and pregnancy. Rev. Obstet. Gynecol..

[143] Devarshi P.P., Grant R.W., Ikonte C.J., Hazels Mitmesser S. (2019). Maternal Omega-3 Nutrition, Placental Transfer and Fetal Brain Development in Gestational Diabetes and Preeclampsia. Nutrients.

[144] Lepretti M., Martucciello S., Burgos Aceves M.A., Putti R., Lionetti L. (2018). Omega-3 Fatty Acids and Insulin Resistance: Focus on the Regulation of Mitochondria and Endoplasmic Reticulum Stress. Nutrients.

[145] Albracht-Schulte K., Kalupahana N.S., Ramalingam L., Wang S., Rahman S.M., Robert-McComb J., Moustaid-Moussa N. (2018). Omega-3 fatty acids in obesity and metabolic syndrome: A mechanistic update. J. Nutr. Biochem..

[146] Enache R.-M., Profir M., Roşu O.A., Creţoiu S.M., Gaspar B.S. (2024). The Role of Gut Microbiota in the Onset and Progression of Obesity and Associated Comorbidities. Int. J. Mol. Sci..

[147] Gao Y., Li W., Huang X., Lyu Y., Yue C. (2024). Advances in Gut Microbiota-Targeted Therapeutics for Metabolic Syndrome. Microorganisms.

[148] Simão D.O., Vieira V.S., Tosatti J.A.G., Gomes K.B. (2023). Lipids, Gut Microbiota, and the Complex Relationship with Alzheimer’s Disease: A Narrative Review. Nutrients.

[149] Fu Y., Wang Y., Gao H., Li D., Jiang R., Ge L., Tong C., Xu K. (2021). Associations among Dietary Omega-3 Polyunsaturated Fatty Acids, the Gut Microbiota, and Intestinal Immunity. Mediators Inflamm..

[150] Matar A., Damianos J.A., Jencks K.J., Camilleri M. (2024). Intestinal Barrier Impairment, Preservation, and Repair: An Update. Nutrients.

[151] Di Tommaso N., Gasbarrini A., Ponziani F.R. (2021). Intestinal Barrier in Human Health and Disease. Int. J. Environ. Res. Public Health.

